# Isolation of a panel of ultra-potent human antibodies neutralizing SARS-CoV-2 and viral variants of concern

**DOI:** 10.1038/s41421-021-00340-8

**Published:** 2021-10-19

**Authors:** Andrey A. Gorchakov, Sergey V. Kulemzin, Sergey V. Guselnikov, Konstantin O. Baranov, Tatyana N. Belovezhets, Ludmila V. Mechetina, Olga Yu. Volkova, Alexander M. Najakshin, Nikolai A. Chikaev, Anton N. Chikaev, Pavel P. Solodkov, Victor F. Larichev, Marina A. Gulyaeva, Alexander G. Markhaev, Yulia V. Kononova, Alexander Yu. Alekseyev, Alexander M. Shestopalov, Gaukhar M. Yusubalieva, Tatiana V. Klypa, Alexander V. Ivanov, Vladimir T. Valuev-Elliston, Vladimir P. Baklaushev, Alexander V. Taranin

**Affiliations:** 1 Institute of Molecular and Cellular Biology, Siberian Branch of the Russian Academy of Sciences, Novosibirsk, Russia; 2 Novosibirsk State University, Novosibirsk, Russia; 3 National Research Center of Epidemiology and Microbiology named after the honorary academician N.F. Gamaleya of the Ministry of Health of the Russian Federation, Moscow, Russia; 4 Federal Research Center of Fundamental and Translational Medicine, Novosibirsk, Russia; 5 Dagestan State University, Makhachkala, Republic of Dagestan Russia; 6 Federal Research and Clinical Center for Specialized Medical Care, FMBA of Russia, Moscow, Russia; 7 Center for Precision Genome Editing and Genetic Technologies for Biomedicine, Engelhardt Institute of Molecular Biology, Russian Academy of Sciences, Moscow, Russia

**Keywords:** Biological techniques, Immunology

## Abstract

In the absence of virus-targeting small-molecule drugs approved for the treatment and prevention of COVID-19, broadening the repertoire of potent SARS-CoV-2-neutralizing antibodies represents an important area of research in response to the ongoing pandemic. Systematic analysis of such antibodies and their combinations can be particularly instrumental for identification of candidates that may prove resistant to the emerging viral escape variants. Here, we isolated a panel of 23 RBD-specific human monoclonal antibodies from the B cells of convalescent patients. A surprisingly large proportion of such antibodies displayed potent virus-neutralizing activity both in vitro and in vivo. Four of the isolated nAbs can be categorized as ultrapotent with an apparent IC_100_ below 16 ng/mL. We show that individual nAbs as well as dual combinations thereof retain activity against currently circulating SARS-CoV-2 variants of concern (such as B.1.1.7, B.1.351, B.1.617, and C.37), as well as against other viral variants. When used as a prophylactics or therapeutics, these nAbs could potently suppress viral replication and prevent lung pathology in SARS-CoV-2-infected hamsters. Our data contribute to the rational development of oligoclonal therapeutic nAb cocktails mitigating the risk of SARS-CoV-2 escape.

## Introduction

Virus-neutralizing monoclonal antibodies (nAbs) have invariably been at the forefront of antiviral therapeutic and prophylactic measures due to their excellent safety and efficacy profiles. Since the very beginning of the pandemic caused by SARS-CoV-2, massive research efforts have been focused on the design of vaccines, as well as the development of antiviral molecules and patient-centered therapeutic modalities. Potent protection afforded by the various vaccine platforms demonstrated in key clinical trials^1–4^ has stimulated global immunization campaigns aiming to prevent COVID-19 and curb the pandemic. Despite this important progress, there is significant need for SARS-CoV-2-neutralizing monoclonal antibodies, as numerous vulnerable categories of individuals either cannot be vaccinated for medical reasons or are unable to mount vaccine-induced protective immune responses. Isolation of SARS-CoV-2-specific nAbs has so far relied on several approaches and included sequencing of individual B cells from convalescent donors or humanized mice^5–24^, SARS survivors^25–27^, as well as screening of antibody, scFv, Fab or VH libraries^28–36^. Hundreds of SARS-CoV-2-neutralizing antibodies of different breadth and potency have already been isolated. Currently, dozens of the lead candidates are undergoing clinical trials^37^, and several antibodies or antibody combinations have received either emergency use authorization or were granted approval from the U.S. Food and Drug Administration (FDA). Significant efforts are now centered on the isolation of potent broadly neutralizing monoclonal antibodies that are also active against other sarbecoviruses, and may therefore be effective against possible escape variants of SARS-CoV-2^38,39^. The emergence of SARS-CoV-2 variants of concern (VOCs) has immediately raised the question of the effectiveness of nAbs against these viral variants. Unfortunately, not all monoclonal antibodies capable of neutralizing the ancestral Wuhan or D614G strains are effective against the VOCs. For example, bamlanivimab almost lost its neutralizing activity against the delta variant^40^. Bamlanivimab and etesivimab fail to neutralize the beta variant^41^, whereas the potency of casirivimab is reduced. However, imdevimab remains effective against alpha, beta, and delta VOCs^40,41^ and sotrovimab retains potency against alpha, beta, and gamma^42^. The efficacy of regdanvimab against the beta variant is significantly reduced, however, in in vivo models, this antibody is still capable of exerting a therapeutic effect^43^. Therefore, isolation and comprehensive characterization of additional SARS-CoV-2-neutralizing monoclonal antibodies continue to be an important area of research, as it opens the opportunity to formulate the cocktails of nAbs that will neutralize or reduce transmission of emerging viral variants that would otherwise escape neutralization by single-antibody preparations.

Here, we isolated a panel of human nAbs specific for SARS-CoV-2 receptor-binding domain (RBD) and characterized their activity in vitro and in vivo. Of these, four nAbs can be classified as ultrapotent. We find that the ACE2 (Angiotensin-Converting Enzyme 2)/RBD interface encompasses a continuum of differentially overlapping epitopes for nAbs that frequently display complimentary profiles of sensitivity to SARS-CoV-2 Spike (S) variants. This informs the rational design of a broader repertoire of RBD-specific cocktails composed of non-competing nAbs that should retain activity against currently circulating viral lineages as well as future variants.

## Results

### Selection of convalescent COVID-19 patients with potently neutralizing sera

**Fig. 1:**
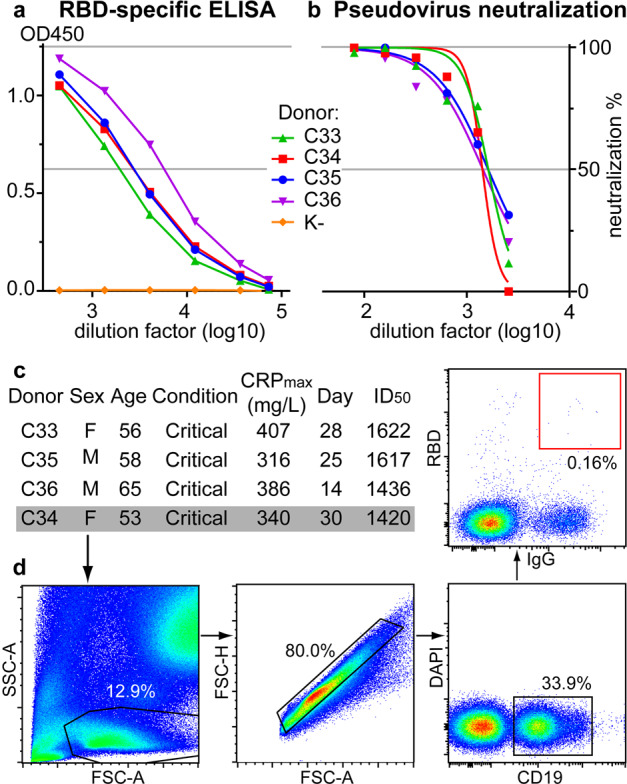
Donor selection and sorting of RBD-specific B lymphocytes from the blood of convalescent COVID-19 patients. **a**, **b** Sera of four select donors were characterized by ELISA for anti-RBD binding potency (**a**) and pseudovirus neutralization activity (**b**). K- stands for a healthy donor. **c** Short medical information for these donors. **d** Gating strategy used for selection of individual RBD+IgG+ B lymphocytes, shown are the representative FACS plots for donor C34.

Two-to-four weeks after the symptom onset, 650 patients with a previously RT-qPCR (Reverse Transcription quantitative Polymerase Chain Reaction)-confirmed SARS-CoV-2 infection were recruited to our study. Blood samples were pre-screened for the presence of RBD-specific antibodies, as these have been demonstrated to correlate with the neutralizing activity of the sera^44^. Consistent with earlier findings on the positive correlation between neutralizing titers and severity of COVID-19^45,46^, sera from four critically ill patients displayed particularly high RBD-specific titers (> 3.6 × 10^4^) as well as potent pseudovirus-neutralizing activity (ID_50_ range 1420–1622) (Fig. 1a, b). These patients were selected for blood donation (Fig. 1c).

### Isolation of RBD-specific human B cells and monoclonal antibodies

CD19^+^ IgG^+^ B cells were isolated from peripheral blood samples and stained using a biotinylated SARS-CoV-2 RBD. To set a stringent gating threshold, B cells from a healthy donor were used as a control. The frequency of CD19^+^IgG^+^RBD^+^ B cells in patients С33, С34, С35, and С36 varied from 0.032 to 0.091% (Supplementary Table S1). Only RBD^high^ B cells were flow-sorted into individual tubes (Fig. 1d). Seventy-two individual B cells were flow-sorted, half of which (40) were from patient C34.

**Fig. 2:**
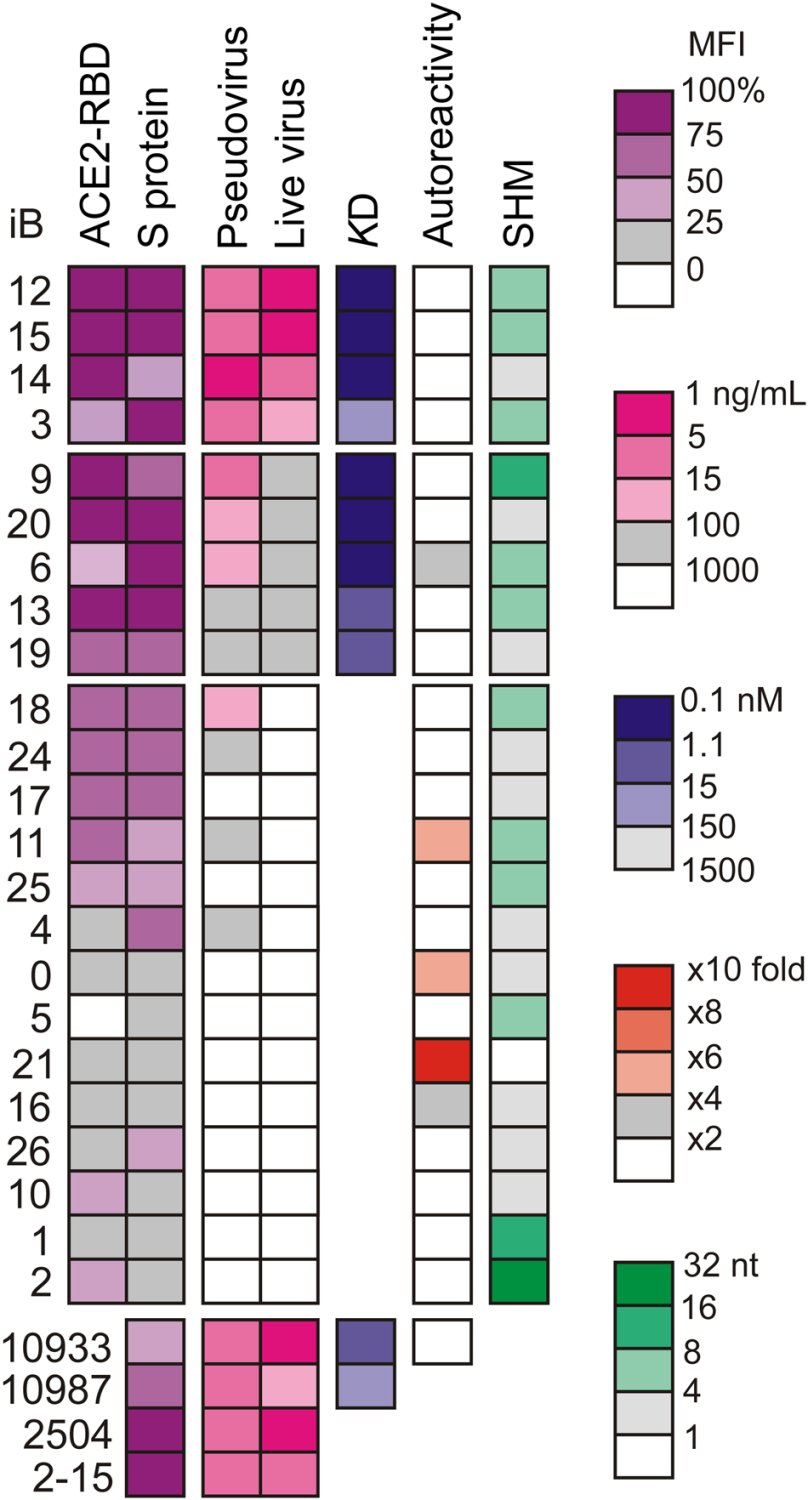
Comparative characteristics of the SARS-CoV-2 RBD-binding human monoclonal antibodies. Heat maps are shown for monoclonal antibody blocking of ACE2/RBD interaction (%), staining of cell-surface expressed SARS-CoV-2 Spike (%), neutralization of Spike-pseudotyped lentiviral particles (IC_50_) or authentic SARS-CoV-2 virus (IC_100_), *K*D for the interaction with recombinant RBD (BLI measurements), autoreactivity (fold MFI increase above background), and SHM rate (number of nucleotide substitutions). Antibodies are grouped and ordered by neutralization potency and were either obtained in this study (iB0-26) or reported earlier (10933, REGN10933; 10987, REGN10987; 2504, COV2-2504; 2015, COVA2-15)^5,8,11^. All numerical values can be found in Supplementary Table S2.

Next, pairs of expressed VH- and VL-gene sequences were successfully retrieved for 33 B cells (1, 3, 4, and 25 B cells from patients C36, C33, C35, and C34, respectively). Of these, 28 had unique combinations of VH and VL sequences. Twenty-five of these sequences were observed in only one cell each, whereas the remaining eight were found across the clones composed of 2 or 3 cells each (iB1, iB2, and iB8). Thus, 28 recombinant IgG1-class antibodies were produced in HEK293T cells and purified via Protein A chromatography. Of these, 23 antibodies turned out to be specific for RBD, as assayed by ELISA (Enzyme-Linked Immunosorbent Assay) (iB7, iB8, iB22, iB23, and iB27 were not followed up, as they lacked appreciable binding to RBD). BLI (Bio-Layer Interferometry) analysis indicated that only two RBD-specific antibodies, iB1 and iB2, were low-affinity (*K*D = 1.0 and 1.5 μM, respectively) and three were in the moderate affinity subgroup (iB3 78 nM, iB4 31 nM, iB10 106 nM). In total, 18 high-affinity RBD-specific antibodies were obtained with a *K*D ranging from 0.47 to 13.3 nM (Fig. 2; Supplementary Fig. S1 and Table S2). We asked whether the above 23 RBD-specific antibodies were reactive towards RBD in the native context of the SARS-CoV-2 S protein. To address this question, FACS (Fluorescence-Activated Cell Sorting) analysis of HEK293T cells expressing SARS-CoV-2 S ptotein on their surface was performed. All the 23 antibodies stained S-expressing HEK293T cells, though the intensity of staining varied broadly (Fig. 2). Notably, none of the antibodies recognized the S proteins from two distantly related alphacoronaviruses, HCoV-229E and HCoV-NL63 (Supplementary Fig. S2), consistent with high degree of sequence divergence of their RBDs and the B cell selection strategy.

In light of the possible therapeutic application, we wished to explore the possibility that the antibodies isolated could be autoreactive, i.e., recognize human self-antigens, as this has previously been reported for a number of SARS-CoV-2-specific antibodies isolated from COVID-19 patients^19,20^. Autoreactivity potential was assessed using a FACS-based HEp-2 immunostaining assay, where only three antibodies (iB0, iB11, and iB21) were scored positive (Fig. 2). None of these have subsequently been shown to possess neutralizing activity (see below).

### Identification of human monoclonal antibodies displaying virus-neutralizing activity

Although several mechanisms of SARS-CoV-2 neutralization by monoclonal antibodies have been described, most of the RBD-specific antibodies do so by disrupting the interaction between RBD and ACE2, the major entry receptor of the virus. Sixteen of the antibodies in our panel were capable of efficiently blocking the interaction between soluble biotinylated SARS-CoV-2 RBD and ACE2 that was ectopically expressed on the surface of HEK293T cells. In this FACS assay, which served as a simple surrogate for virus-neutralization test, nine antibodies were shown to potently block ACE2/RBD interaction (> 75% MFI (mean fluorescence intensity) reduction) (Fig. 2).

**Fig. 3:**
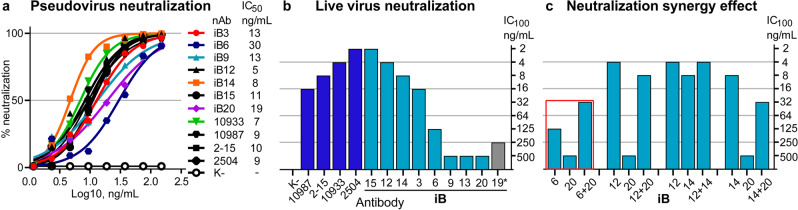
In vitro virus neutralization by the SARS-CoV-2 nAbs. Pseudovirus (**a**) or live (**b**, **c**) SARS-CoV-2 virus were used. nAbs were either published before (dark blue columns) or obtained in this study (cyan). iB19* neutralization was incomplete (gray). glVRC01 is an irrelevant HIV-specific antibody. 10933, REGN10933; 10987, REGN10987; 2-15, COVA2-15; 2504, COV2-2504. Antibody pair (iB6 + iB20) displaying synergistic activity is marked by a red frame (**c**).

Next, we carried out an analysis of virus neutralization using S-pseudotyped lentiviral particles and ACE2-HEK293T cells as the targets. At the cut-off threshold of IC_50_ below 1 μg/mL, 13 antibodies demonstrated virus-neutralizing activity (Fig. 2; Supplementary Table S2), of which 7 most promising nAbs were identified as neutralizing at IC_50_ below 0.03 μg/mL (Fig. 3a).

In order to understand whether the antibodies were active towards the authentic SARS-CoV-2, virus neutralization assays were performed using authentic SARS-CoV-2 and VeroE6a cells (Fig. 3b; Supplementary Table S2). Neutralization activity was indeed confirmed, and four of the leading nAb candidates—iB15, iB12, iB14, and iB3—were considered ultrapotent (arbitrary cut-off threshold of IC_100_ ≤ 16 ng/mL). Accordingly, our BLI analysis showed that except for iB3 (*K*D = 77.8 nM) all of these nAbs had high affinity to RBD with a *K*D ranging from 0.47 to 1.09 nM (Fig. 2; Supplementary Fig. S1 and Table S2). To explore whether synergy between iB-series nAbs may exist, various antibody combinations were tested in live virus neutralization assay. Of these, only the cocktail made of nAbs iB6 and iB20 targeting non-overlapping epitopes (see below) was shown to display detectable synergy (Fig. 3c).

### SARS-CoV-2-neutralizing antibodies perform on par with the published top neutralizing antibodies

Neutralization assays performed by different groups may suffer inconsistencies due to minor details in virus production, handling, and assay readout. To avoid these possible pitfalls we decided to compare the antibodies in the same setting. Specifically, we asked whether the antibodies isolated in our work would display neutralization activities that compare well to the four of the most potent RBD-specific antibodies, two of which have already advanced to clinical use. To this end, we produced highly potent SARS-CoV-2-neutralizing antibodies REGN10933 (casirivimab), REGN10897 (imdevimab)^8^, COVA2-15^5^, and COV2-2504^47^. In our hands, these antibodies displayed an IC_50_ of 7, 9, 10, and 9 ng/mL against the S-pseudotyped lentivirus (Fig. 3a), and IC_100_ of 4, 16, 8, and 2 ng/mL against the authentic SARS-CoV-2, respectively (Fig. 3b). These numbers are in line with the published values, and minor differences are likely attributable to the differences in assay set-up. Thus, the lead four monoclonal antibodies obtained in our work display neutralization potency in the range of the best SARS-CoV-2-neutralizing RBD-specific antibodies published to date.

### The isolated RBD-specific SARS-CoV-2-neutralizing antibodies belong to public clonotypes

Previously, significant diversity of genes encoding RBD-specific SARS-CoV-2-neutralizing antibodies has been reported, with extensive enrichment in antibodies having nearly germline configuration^5,19,48^. We observed a similar trend, as representatives of 10 VH- and 7 VL- gene segments were present among the antibody-encoding genes (Supplementary Figs. S3, S4 and Table S3). Consistent with the previous findings on the enrichment of particular classes of RBD-specific nAbs, several VH genes, such as VH3-53 (iB18, iB20, iB24, iB25), VH1-2 (iB3, iB12), VH1-69 (iB6, iB9), VH1-58 (iB14), and VH3-66 (iB19) were present in our panel. Notably, VH3-53/66-derived nAbs have short (11–13 residues) CDRH3s (Heavy chain Complementarity Determining Regions 3) and may be attributed to the recurrent class I antibodies known to bind a common epitope in “up” RBD conformation^49,50^. VH1-58/VK3-20-encoded iB14 appears to belong to another public clonotype that binds to convex tip of RBD^51^, COV2-2196^11^, and 2C08^52^.

We next turned to the analysis of SHM (Somatic Hypermutation) levels in our antibodies, and only two of these, iB1 and iB2, were encoded by the VH genes displaying high SHM rate (8–10 aa). Notably, these two antibodies had the lowest affinity to RBD and lacked neutralizing activity (Fig. 2; Supplementary Table S3). Four antibodies were derived from nearly germline VH sequences (no amino acid changes). One of these, iB21 showed the highest autoreactivity score (Fig. 2). The remaining antibodies had 1–4 aa substitutions. It was this subgroup of antibodies that had pronounced neutralizing activity in our assays. We asked whether neutralization was in any way related to the antibody affinity to RBD, CDR3L/CDR3H length, or other quantitative features measured in our experiments, and observed that only the MFI of S-expressing cell staining and ability to block the ACE2/RBD interaction were significantly correlated with the neutralization potency (Supplementary Fig. S5).

### BLI competition analysis defines a continuum of partially overlapping antibody epitopes in the RBD-ACE2 interface

We turned to BLI measurements of cross-competition between 22 RBD-specific antibodies for binding to RBD. In these experiments, RBD was immobilized to the sensor and then saturated by the first antibody. The pre-formed RBD-Ab1 complex was then exposed to a competing Ab2. Most of the nAbs fell into one of the two distinct clusters (Supplementary Fig. S6). Cluster I encompassed highly potent antibodies iB3, iB12, iB14, and iB15, as well as moderately potent iB13 and weakly potent iB11. Cluster II was formed by the moderately and weakly potent iB9, iB17, iB18, iB19, iB20, iB24, and iB25. One more neutralizing antibody, iB6, branched distinctly. Non-neutralizing antibodies clustered separately from the nAbs.

In order to understand whether the antibodies isolated in our study could be combined together as well as with several published nAbs to form cocktails, we proceeded to compare the epitope specificity of our 9 leading candidates with REGN10933, REGN10987, COVA2-15, and COV2-2504^5,8,11^. REGN10933 and REGN10987 are known to recognize non-overlapping epitopes on RBD, with the epitope of the former antibody found directly at the ACE2/RBD interface, and the epitope of the latter antibody found at the edge. The structures of COVA2-15 and COV2-2504 epitopes have not been determined with high resolution. Yet, it is known that both antibodies compete with ACE2 for binding to RBD and that COVA2-15 interacts with RBD in both “up” and “down” conformations^5^.

**Fig. 4:**
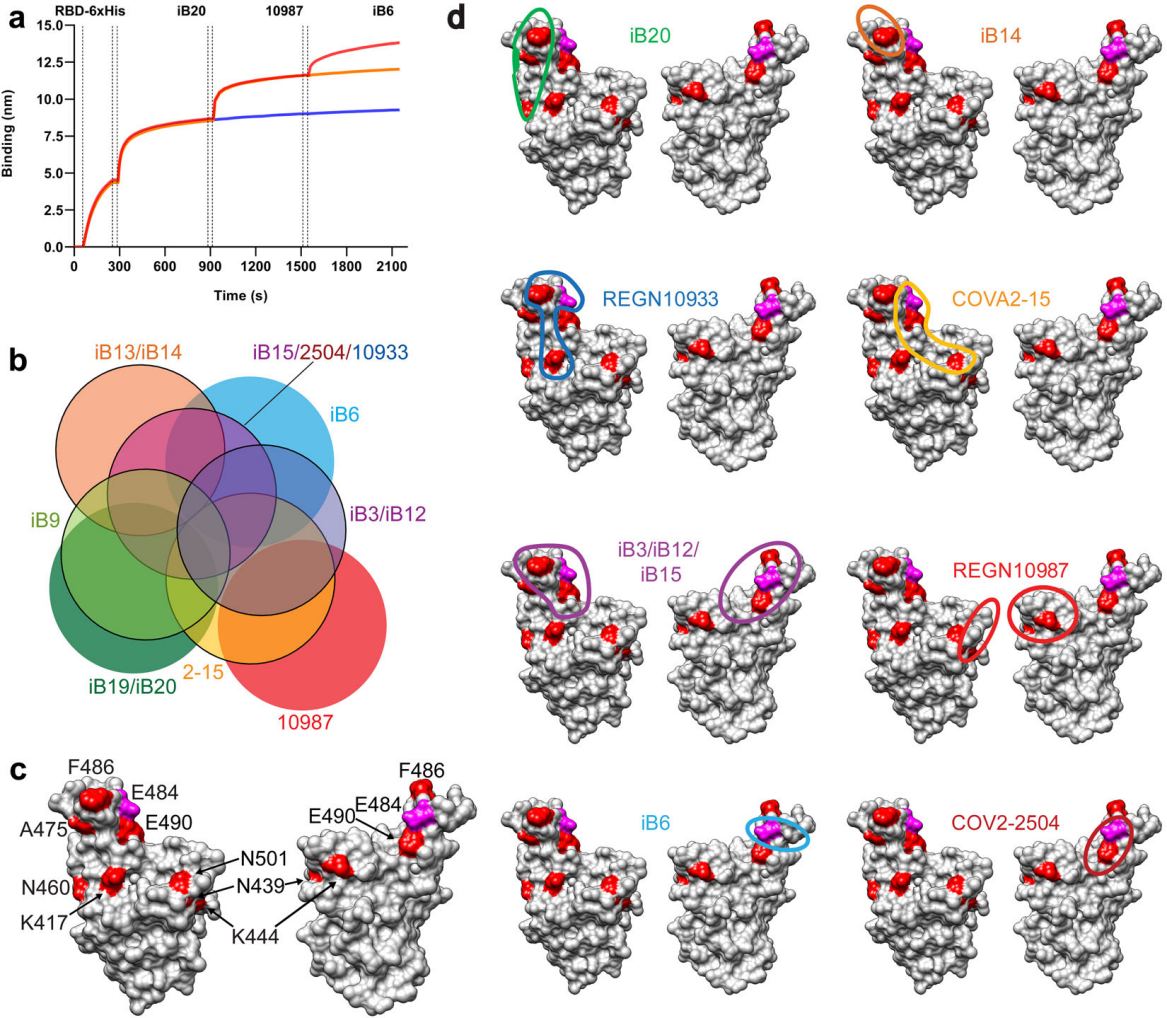
Epitope binning of SARS-CoV-2 RBD-specific nAbs. The analysis includes either previously published (REGN10987, REGN10933, COVA2-15) or iB-series of nAbs. **a** BLI analysis of competition between nAbs iB20, REGN10987, and iB6 for RBD binding. **b** Venn diagram showing mutual overlap between the tentative epitopes of nAbs forming eight distinct bins. The diagram was plotted based on the BLI data (Supplementary Figs. S6 and S7). **c** Positions of nine mutations escaping neutralization by at least one of the tested antibodies. Residues mutated are highlighted red or pink on the 3D model of SARS-CoV-2 RBD. **d** Tentative positions of nAb footprints are circled.

Our BLI experiments showed that iB20, iB6, and REGN10987 can bind simultaneously to RBD, indicating that the ACE2/RBD-interface region encompasses three distinct, non-overlapping epitopes (Fig. 4a). The rest of the nAbs recognize a continuum of epitopes that display various degrees of overlap with the above three epitopes (Fig. 4b; Supplementary Figs. S6 and S7). For instance, the antibodies iB3, iB12, iB13, iB14, iB15, REGN10933, and COV2-2504 fully compete with each other but show different competition patterns when tested against СOVA2-15 and REGN10987. The latter pair competes with iB3 and iB12 but not with iB13 and iB14. Three other antibodies, REGN10933, iB15, and COV2-2504, do not compete with REGN10987 but strongly compete with COVA2-15. Antibodies iB3, iB12, iB13, iB14, iB15, REGN10933, СOV2-2504, and COVA2-15, when pre-bound to RBD, ablate binding of the iB9, iB19, and iB20. Yet, in the reverse set-up of the assay, the blocking was incomplete. To summarize, our competition analysis so far is consistent with the clustering of the 13 nAbs tested into 8 epitope specificity groups: iB13/iB14, REGN10933/COV2-2504/iB15, iB3/iB12, iB9, COVA2-15, iB19/iB20, iB6, and REGN10987 (Fig. 4b). Interestingly, this continuum of epitopes provides multiple opportunities to formulate cocktails of noncompeting nAbs. Even in our limited panel of the tested nAbs, seven such cocktails may be designed in addition to the clinical-stage combination REGN10933 + REGN10987. These include iB14 + COVA2-15, iB14 + REGN10987, iB20 + iB6, iB15 + REGN10987, COV2-2504 + REGN10987, iB20 + REGN10987, and finally a tripartite cocktail iB6 + iB20 + REGN10987.

### Both published and newly identified nAbs differ in their sensitivity to select SARS-CoV-2 mutations, including those present in the VOCs

SARS-CoV-2 is known to accumulate genetic changes throughout the pandemic, and so far the mutation rate has been relatively low. However, the emergence of viral lineages resistant to the monoclonal antibodies or polyclonal sera has already been reported and is of great concern. To explore whether iB-series antibodies are sensitive to the RBD mutations, we established a panel of 9 variants of S protein, each carrying a distinct single-amino acid substitution in RBD (K417N, N439K, K444Q, N460T, A475V, Е484K, F486K, F490L, and N501Y) and tested it in pseudovirus neutralization assay against seven nAbs (iB3, iB6, iB9, iB12, iB14, iB15, and iB20) and four benchmark nAbs (REGN10987, REGN10933, COVA2-15, COV2-2504). Notably, N501Y substitution is present in the RBD of several viral VOCs, namely in the 501Y.V1 (B.1.1.7 lineage), 501Y.V2 (B.1.351 lineage), and 501Y.V3 (P.1 lineage). In the latter two, N501Y is found in combination with K417N and E484K. The E484K mutation was also found in the spreading B.1.525 lineage and was demonstrated to ablate the activity of a number of nAbs^7,8,49,53–56^.

**Fig. 5:**
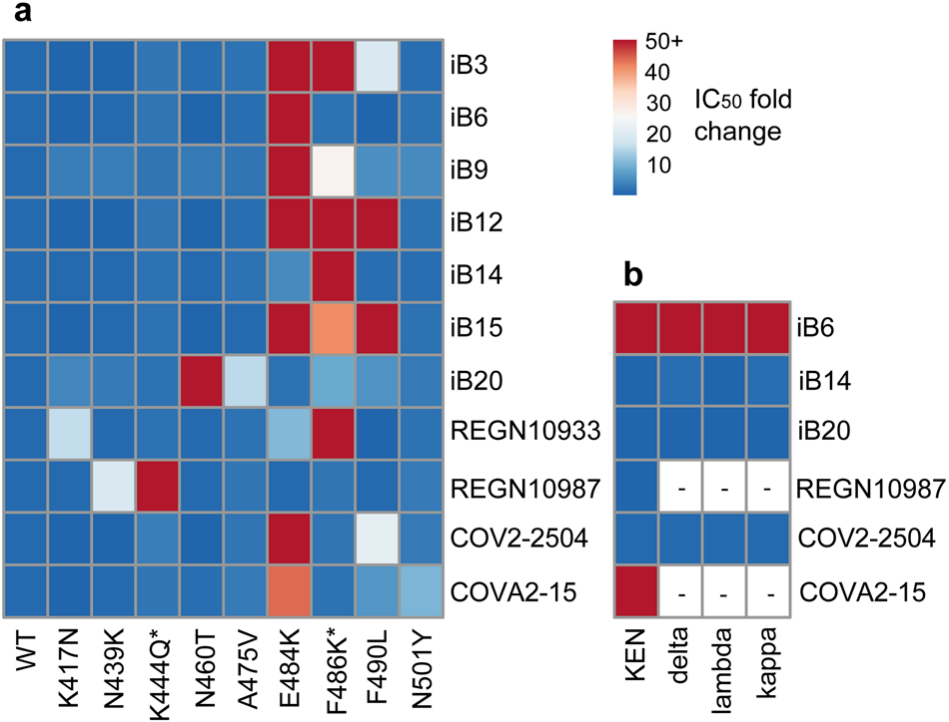
Neutralization profiles for the iB-series of nAbs as well as for some of the previously reported reference nAbs tested against WT and selected amino acid residue mutant pseudoviruses (positions 406–501 aa). **a** Heatmap displaying nAb IC_50_ fold change, when tested against a single amino acid mutant virus relative to WT. Asterisks denote mutations that are absent from the currently circulating viral isolates and which have been shown to confer resistance to some of the clinical-stage nAbs. **b** Heatmap displaying nAb sensitivity to double and triple mutations in the RBD, which correspond to the substitutions found in several currently circulating VOCs.

Our data indicate that of these S variants, three mutations, namely Е484K, F486K, and F490L had the most pronounced effect on the neutralizing ability of nAbs (Fig. 5a), which is in agreement with above studies. Specifically, the E484K S variant was completely resistant to 7 out of the 11 nAbs tested. Three nAbs, iB14, iB20, and REGN10987, were not affected, whereas REGN10933 was 11-fold less potent against this mutant. F490L fully abrogated neutralization by iB12 and iB15. This variant reduced virus-neutralizing activity of iB3 and COV2-2504 by ~20 fold, whereas iB9, iB20, and COVA2-15 were only marginally affected. Activity of iB20 was strongly attenuated by N460T and A475V. Consistent with the published data, K417N substitution reduced the neutralizing activity of REGN10933, whereas N439K and K444Q affected REGN10987^54,55^. None of the latter three mutations appreciably affected any of the iB-series of nAbs. COVA2-15 was the only nAb in this panel that was somewhat sensitive to the N501Y S variant, which is in line with the recent findings^57^.

To refine the interplay between the epitopes recognized by nAbs isolated in our study as well as by the reference nAbs, the positions of the tested RBD substitutions were placed on the 3D structure of RBD (7CH5)^58^ (Fig. 4c, d). As expected, the key residues important for neutralization by each antibody clustered together. This clustering matches very well both the known epitope positions for REGN10933 and REGN10987^5,8^ and the Venn diagram based on our competition data (Fig. 4b). Thus, the escape mutation patterns observed further substantiate the design of seven competition-based nAb cocktails proposed above.

Next, we asked whether our lead nAbs iB14 and iB20 would be active against lentiviral particles pseudotyped with the triple-mutant S carrying the substitutions at key residues K417, E484, and N501 (KEN), given that this mutant combination is present in the RBD of two viral VOCs, 501Y.V2 and 501Y.V3. In line with the inability of individual S mutants to confer resistance to iB14 and iB20, as well as to the reference nAb REGN10987, these three nAbs were found to retain full neutralizing activity, unlike iB6 and COVA2-15, whose activity was abolished (Fig. 5b). As expected, the cocktails of iB6 with iB20 or REGN10987, as well as of COVA2-15 with iB14 were fully efficient in neutralizing the KEN variant (Supplementary Fig. S8).

Finally, to extend our studies, we assessed the ability of iB6, iB14, and iB20 to neutralize lentiviral particles pseudotyped with S variants each carrying two RBD substitutions found in the delta (B.1.617.2 (L452R + T478K)), kappa (B.1.617.1 (L452R + E484Q)), and lambda (C.37 (L452Q + F490S)) viral lineages that have appeared in circulation very recently. nAbs iB14 and iB20 retain full neutralization potency against these variants (Fig. 5b) unlike iB6, which appears sensitive to the substitutions at L452 residue shared between the above mutant S proteins. Taken together, these results point to the possible utility of iB14 and iB20 for therapeutic use in patients infected with such SARS-CoV-2 variants.

### iB-series of antibodies display potent in vivo activity

**Fig. 6:**
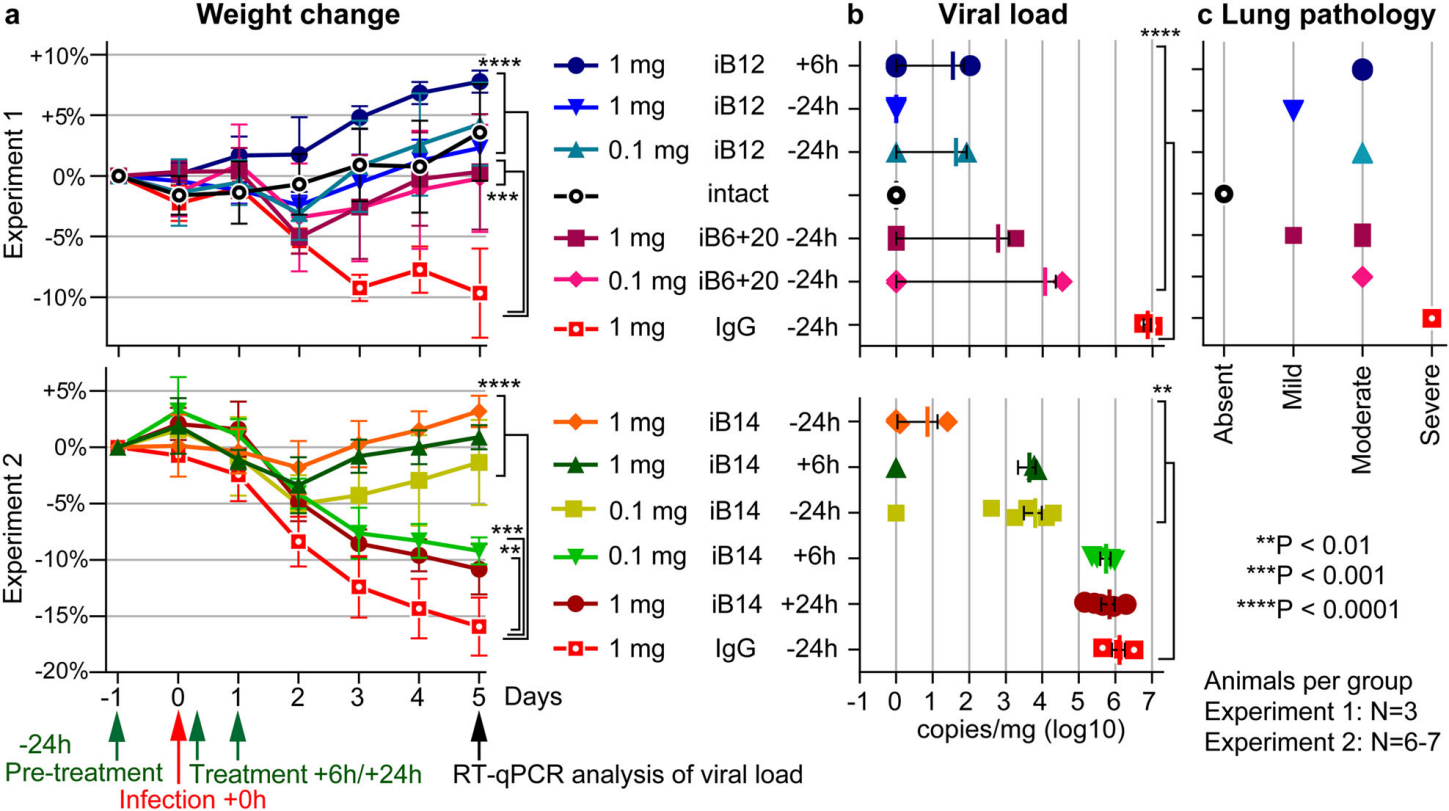
Efficacy of iB12, iB14, and iB6 + iB20 cocktail in treatment and prophylaxis of SARS-CoV-2 infection in a hamster model. In a prophylactic regimen, antibodies were administered i/p 1 day before infection at a dose of 1 or 0.1 mg/animal (i.e., ~10 or 1 mg/kg weight). Control animals received 1 mg of total human IgG. In the therapeutic regimen, iB12 antibody (1 mg/animal) was given 6 h following the viral challenge (Experiment 1), whereas iB14 antibody was injected either 6 h (0.1 or 1 mg/animal) or 24 h (1 mg/animal) following the viral challenge (Experiment 2). Intact animals formed a separate control group. Animals were sacrificed on day 5 following infection. Lungs were weighted and used for RT-qPCR quantification of the viral load. **a** Relative weight dynamics in the prophylaxis and treatment groups following administration of iB nAbs or control total human IgG. Error bars represent means ± SD. **b** Impact of iB nAbs on the levels of viral transcripts in hamster lungs (RT-qPCR with *RdRp* primers). RT-qPCR with *E* gene-specific primers produced very similar results (not shown). Animals with strongly reduced human nAb titers in the blood were considered as outliers due to the technical error of nAb injection and were excluded from the analysis. Error bars represent means ± SEM. **c** Lung pathology scores for hamster lungs at 5 dpi (Supplementary Fig. S9).

Whether the in vitro virus-neutralizing activity of iB-series antibodies translates into in vivo activity was unknown, and we proceeded to test this in a Syrian hamster infection model. Specifically, we first selected iB12 and a 1:1 mixture of iB6/B20 to be used as a prophylactics and/or therapeutics of SARS-CoV-2 infection and pathology in hamsters (Fig. 6, Experiment 1). After the completion of this experiment, several SARS-CoV-2 VOCs had emerged, which were predicted to be resistant to iB12 in our above analysis, so we conducted an additional in vivo experiment with the iB14 antibody exhibiting a favorable profile of sensitivity to the viral variants (Fig. 6, Experiment 2).

Both prophylactic and therapeutic regimens were clearly efficacious. Whereas control animals lost up to 15% of their weight by 5 dpi (day post infection), all the animals from experimental groups gained weight from 3 dpi (Fig. 6a). Quantification of viral RNA in lung tissue homogenates indicated that hamsters who were given the neutralizing antibodies prophylactically had overall 2–6 orders of magnitude lower levels of viral RNA, compared to the controls. Notably, therapy of the infected hamsters with iB12 or iB14 at the dose of 1 mg was also protective, as viral load was dramatically reduced (Fig. 6b). In the control animals, in addition to the high viral load, all the signs of interstitial pneumonia were found, including massive peribronchial and perivascular leucocyte infiltration accompanied by edema, multifocal necrosis in interstitium and significant pulmonary consolidation that affected more than 50% of the lung. Prophylactic administration of iB12 (1 mg) had the strongest protective effect in the lungs, as only minimal lung damage was found in this group. Lower dose of iB12 (0.1 mg), the iB6 + iB20 mix (0.1 and 1 mg) as well as the therapeutic injection of iB12 prevented the development of pneumonia, although thickened alveolar septae with mild type II alveolar epithelial cell hyperplasia or desquamated epithelium in the bronchiolar lumena and perivascular cuffing were occasionally observed (Fig. 6c; Supplementary Fig. S9). Therapy with a low dose of iB14 or delayed administration of iB14 did not appreciably reduce viral RNA concentration in hamster lungs, although the weight loss was significantly lower.

## Discussion

Mounting evidence suggests that emerging viral variants tend to display resistance to neutralization not only to current clinical-stage monoclonal antibodies, but also to sera from convalescent patients and immunized donors^54,59–62^. Here, we used single B cell sorting from four severely ill COVID-19 patients who got infected early in the course of the pandemic to isolate human SARS-CoV-2-specific nAbs. In total, a panel of 23 antibodies was obtained, of which 13 were virus-neutralizing and 4 could be classified as ultrapotent. We focused on seven lead candidates (iB3, iB6, iB9, iB12, iB14, iB15, iB20) to explore their ability to neutralize several viral variants. Notably, both naturally occurring and synthetic mutants absent from the circulation were included in our analysis, as these have been demonstrated to escape neutralization by one or more clinical-stage nAbs^59,63^. Our data indicate that while each of the nAbs tested was ineffective against at least one of the S mutants, none of the mutations ablated the activity of the entire panel of nAbs. Next, based on the sensitivity profiles of the lead nAbs, we narrowed the selection of candidates to neutralize the “worst case scenario” viral VOC, B.1.351 defined by the triple substitution (KEN) within the RBD. In this analysis, two nAbs, iB14 and iB20 were found to retain full neutralizing activity, consistent with the failure of single-amino acid S mutants K417N, E484K, and N501Y to confer resistance to these nAbs. Of relevance, iB14 and iB20 were also found to be fully active against pseudotyped lentiviral particles with RBD substitutions in the Spike corresponding to the delta, kappa, and lambda viral lineages. Interestingly, one more residue, F486, has been reported to be critical for binding of several potent nAbs, including the clinical-stage REGN10933, LY-CoV555, and LY-CoV016^54,55,63^. In our panel, iB6 could fully neutralize the mutant pseudovirus bearing the F486K substitution. Thus, from the practical standpoint, our panel encompasses three nAbs—iB6, iB14, and iB20 that complement the repertoire of the published nAbs in terms of activity against the most pressing S mutations.

Therefore, based on these nAbs, one can envisage the design of two therapeutic cocktails such as iB6 + iB20 and iB6 + iB14. Importantly, iB6 and iB20, unlike iB6 and iB14, do not compete for binding to S protein, i.e., they recognize non-overlapping epitopes, and this cocktail (iB6 + iB20) represents an optimal combination as it should provide a broader neutralization coverage of the emerging and future viral variants. To analyze whether the cocktail composed of iB6 and iB20 performs well in vivo as a prophylactic agent, we had it tested in a hamster infection model, and confirmed that this was indeed the case.

Yet, another important feature that may affect clinical performance of the antiviral nAbs is their potency, as it could translate into reduced dosage and manufacturing costs^64^. We turned to one of the most potent nAbs in our panel, iB12, to test it as both prophylactic and therapeutic agent in hamsters, even though it is sensitive to substitutions at key E484, F486 and F490 residues of the Spike. When faced against the D614G-bearing isolate of SARS-CoV-2, iB12 was fully functional and suppressed both viral replication and lung pathology in the infected animals.

From a broader perspective, our data on the resistance to viral escape mutations and epitope binning open an exciting opportunity to formulate nAb cocktails with a number of clinical-stage nAbs. For instance, iB6 and iB20 could be combined with REGN10987 to form a tripartite cocktail composed of entirely non-overlapping nAbs targeting the ACE2/RBD interface. Other interesting combinations are also possible, such as iB14 + COVA2-15 or iB14 + COV2-2504, offering the advantage of a broader antibody footprint and lower risk of mutation escape.

There are also some limitations of this study. Syrian hamster model of SARS-CoV-2 infection used in this work does not fully address the complexity of COVID-19 in humans. Only clinical trials will be informative regarding the efficacy and safety of the panel of nAbs obtained in our work in the context of COVID-19 prophylaxis and therapy. To test for viral escape from neutralization by the nAbs, S-pseudotyped lentiviral particles were used, therefore these data may need validation using authentic SARS-CoV-2 viral variants. We did not test the nAb stability or half-life in the bloodstream—the parameters that are critical for subsequent drug development.

Given that nAbs tested in our study were screened against SARS-CoV-2 RBD and were exclusively RBD-specific, we constructed S protein variants corresponding to the viral VOC by incorporating only the mutations found within the RBD, although these variants do contain additional substitutions in the N-terminal domain and S2 regions of the Spike.

## Materials and methods

### Convalescent donor samples

Peripheral blood samples were collected from more than 650 convalescent donors with a laboratory-confirmed COVID-19 (commercial RT-qPCR tests and/or specific pattern of polysegmental viral pneumonia with areas of “ground glass” opacity on high-resolution computed tomography) who were treated at the FRSC FMBA of Russia (Moscow). De-identified heat-inactivated serum samples displaying anti-RBD Spike IgG titers (SARS-CoV-2-IgG-IFA test, Xema Ltd, Russia) 1:5000 or higher were selected for further analyses. Specifically, 12 serum samples were prioritized for RBD-specific ELISA (see below), of which fresh peripheral blood samples were immediately available for four patients. Control samples were obtained from healthy individuals without any history of SARS-CoV-2 infection.

### Expression and purification of SARS-CoV-2 RBD-His6 antigen

A plasmid pCAAGS-mRBD-His6 encoding a His-tagged RBD of SARS-CoV-2 S protein was a kind gift of Prof. Florian Krammer^65^. HEK293T cells were cultured in DMEM supplemented with 10% FBS and 2 mM glutamine at 37 °C in 5% CO_2_. For transfection, they were seeded onto four 100 mm dishes at 70% density, and 18 h later transfected with 12 µg/dish of the plasmid pCAAG-mRBD-His6 premixed with 24 µL/dish of the Lipofectamine 3000 reagent (Invitrogen) according to the manufacturer’s instruction. Twenty-four hours later the cells were harvested with trypsin-EDTA, resuspended in a fresh DMEM medium supplemented with 10% FBS, 2 mM glutamine, and 20 mM HEPES, pH 7.2, and seeded onto 850 cm^2^ roller dish (Corning). Three days later the medium was collected and applied on a 1 × 3 cm Ni-NTA-agarose column (Novagen). The resin was washed with 25 mL of wash buffer (25 mM Tris-HCl, pH 7.5, 500 mM NaCl, 10% (v/v) glycerol, and 1 mM 2-mercaptoethanol), and three more times with a wash buffer supplemented with 10, 30, and 50 mM imidazole (20 mL each). The protein was eluted with the buffer containing 250 mM imidazole. Protein concentration in the fractions was estimated by Bradford reagent, and the fractions with the highest concentrations were combined and dialyzed either against PBS (Phosphate Buffer Saline) for immediate use or against a buffer containing 25 mM Tris-HCl, pH 7.5, 10% glycerol, and 250 mM NaCl, followed by the same buffer with 50% glycerol, and stored at –20 °C.

### RBD biotinylation

RBD-His6 (1 mL, 1 mg/mL in PBS) was reacted with 10 μL Biotin-X-X-NHS (10 mg/mL in DMF) (Merck) for 1 h at room temperature. Once the reaction was complete, the excess of biotin was removed by dialysis against PBS.

### ELISA-based detection of SARS-CoV-2 RBD-specific IgGs in human sera

The antigen (RBD-His6) was diluted in PBS to a final concentration of 0.8 µg/ml, and 50 µL of this solution was applied to each well of a high binding capacity 96-well plate from Costar (Corning® 96 Well EIA/RIA Assay Microplate). After 24 h incubation at 4 °C, the solution was removed, and the plates were blocked with 250 µL of 4% (w/v) nonfat milk (BioRad) in PBS + 0.5% (v/v) Tween-20 (PBST) for 1 h at room temperature. Then the wells were washed three times with PBST, and the pre-diluted blood sera or plasma in PBST with 1% (w/v) non-fat milk was added (100 µL per well). Following incubation at 37 °C for 1 h, the wells were washed six times with PBST. Next, 1:30,000 dilution of a goat anti-human IgG HRP-conjugate (Sigma-Aldrich, cat #A8667) in PBST was added (100 µL/well). After incubation at 37 °C for 40 min, the wells were washed six times with PBST and a Liquid Substrate solution was added (100 µL/well). The Liquid Substrate solution for one 96 plate was prepared by mixing 1 mL of 5 mM TMB (3,3’,5,5’-Tetramethylbenzidine) solution in 30% (v/v) DMSO with 10 mL of 0.023% H_2_O_2_ solution in 31 mM citrate buffer (pH = 4.0). After incubation for 5 min with Liquid Substrate solution, the reaction mixtures were quenched by 0.25 М H_2_SO_4_ (100 µL/well). The signal was quantified by measurement of optical density at 450 nm (OD_450_) using Tecan Spark plate reader. The data were presented as a function of OD_450_ signal vs serum/plasma dilution factor, and the levels of RBD-specific IgG antibodies were expressed as titers (defined as the final dilutions of samples at which the signal is above the background).

### Expression and purification of recombinant ACE2-hFc and SUMOstar-ACE2 fusions

The sequence encoding N-terminal peptidase domain of human ACE2 (residues 18–615) was cloned into pAbVec vector in frame with a C-terminal Fc tag of the human IgG1 (domains CH1-CH3). Also, a construct encoding His6-SUMOstar-ACE2 fusion (ACE2 residues 18–740) was obtained by standard cloning into the pM-SUMOstar mammalian expression vector (cat #7121, LifeSensors). The expression constructs obtained were transiently transfected into HEK293T cells as described above. The supernatants were collected on day 7 post-transfection. Fc-tagged ACE2 protein was then purified with a Protein A column, whereas His6-SUMOstar-ACE2 fusion was purified on a Ni-NTA resin.

### Single B cell sorting

PBMCs (peripheral blood mononuclear cells) were isolated from 9 to 18 mL venous blood by differential centrifugation over Ficoll Paque. Live B cells were identified by staining with DAPI, anti-human CD19-PE (Sorbent, Russia) and anti-human IgG-FITC (Sorbent, Russia) conjugates (5 µL/1 million PBMCs each). B cells were additionally stained with biotinylated recombinant SARS-CoV-2 RBD (2 µg/mL) followed by detection with APC-labeled streptavidin (Thermo Fisher Scientific). Target B cell population was gated as RBDhighCD19+IgG+DAPI^−^. Individual RBD-specific B cells were single sorted into PCR tube strips pre-aliquoted with 10 µL H_2_O and 0.25 µL RiboLock (Thermo Fisher Scientific) using Sony SH800 cell sorter (single-cell mode); the strips were kept frozen at –70 °С.

### Single-cell cDNA synthesis, reverse transcription PCR, and cloning of antibody sequences into expression vectors

All procedures were performed essentially as described^66^, except that the protocol for μ gene amplification was used for γ and λ genes. Primer set reported by Tiller and colleagues^67^ was used for amplification of antibody gene (VHγ, Vκ or Vλ) sequences. Paired amplicons (VHγ + Vκ/Vλ chains) were Sanger-sequenced and cloned into pAbVec series of vectors encoding constant regions of human γ1, κ or λ chains, as described^67^.

### Antibody expression and purification

HEK293T cells were used for antibody expression. Pairs of expression constructs encoding appropriate light and heavy chains were delivered into HEK293T cells using calcium phosphate transfection^68^. Typically, 12 h before transfection 107 cells were plated on a 150 cm^2^ flask in IMDM (Gibco, Thermo Fisher Scientific) supplemented with 10% FBS (Gibco, Thermo Fisher Scientific) and Penicillin/Streptomycin mix (Gibco, Thermo Fisher Scientific). Plasmid DNAs were pre-mixed in a 1:1 molar ratio, and 2 М CaCl_2_ was added to a final concentration of 25 mM. Volume of the transfection mixture was adjusted by adding sterile water to 3 mL. The mixture was slowly added to 3 mL 2× HBS solution (50 mM HEPES, 1.5 mM Na_2_HPO_4_, 280 mM NaCl, 10 mM KCl, 12 mM sucrose, pH = 7.11) under constant vortexing, and the resulting solution was added dropwise to the cells. 8 h later, growth medium was replaced with a serum-free EX-CELL® 293 Serum-Free Medium (Sigma). Transfected cells were kept for 6 days in a CO_2_ incubator at 37 °C, 5% CO_2_. Cell supernatants cleared of the cell debris by brief centrifugation (4000× *g*, 10 min) were filtered through a 0.22 μm PES-filter (TPP), and antibodies were purified on Protein A agarose column (McLab, USA, #PPA-503) according to the manufacturer’s instructions. Briefly, after loading the column was washed twice with PBS. The antibodies were eluted with 0.1 M glycine, pH 2.7, 150 mM NaCl, into the neutralization buffer of 1 M Tris, pH 8.0, in a 1:10 ratio. Purity of antibodies was controlled by running a 15% SDS-PAGE. Antibodies were then dialyzed overnight in PBS and concentrated using Ultra-15 Ultracel-100K (Amicon) to a final concentration of 2 mg/mL.

### Cloning of wild-type (WT) and mutant Spike variants

The construct pCAGGS-S carrying a codon-optimized cassette encoding a full-length SARS-CoV-2 S protein (reference Wuhan-Hu-1 isolate) was first modified to delete the sequence that corresponds to the 19 C-terminal residues of the S protein, which has been shown to boost the viral titers^69^. pCAGGS-SΔ19 plasmid was obtained. Next, sets of complementary mutagenic primers (27 nt each) centered at the desired site were used to introduce the individual mutations (E406W, K417N, N439K, K444Q, N460T, A475V, E484K, F486K, F490L, N501Y), double mutations (L452R + T478K, L452Q + F490S, L452R + E484Q) or a triple “KEN” mutation (K417N + E484K + N501Y) into the coding sequence of SΔ19. Sequence identity was confirmed by Sanger sequencing.

### FACS assay for antibody binding to Spike proteins expressed on the surface of HEK293T cells

Antibodies were tested for how well they may recognize full-length SARS-CoV-2, HCoV-229E, and HCoV-NL63 S proteins expressed on the surface of HEK293Т cells. Cells were transfected with appropriate plasmids 1–2 days before the assay, washed with PBS and incubated with antibodies at the final concentration of 3 µg/mL for 30 min at 4 °С. After three times of washing with PBS, APC-conjugated donkey-anti-human IgG (1:600 dilution) (Jackson ImmunoResearch) was added and incubation proceeded for an additional 30 min. To exclude dead cells, 7AAD (Sigma) was added to a final concentration of 0.5 µg/mL. Cell suspensions were run on a BD FACS Canto II cytometer.

### FACS-based assay for competitive antibody binding to SARS-CoV-2 RBD (ACE2 blocking cell-based assay)

Antibodies (2 μg/mL) were premixed with biotinylated SARS-CoV-2 RBD (0.3 μg/mL) in FACS staining buffer (PBS + 1.5% FBS) and incubated for 15 min at room temperature. Antibody/RBD mixture was added to the suspension of 30,000 ACE2-HEK293T cells in the same buffer and incubation proceeded for 30 min at 4 °С. Next, the cells were washed two times with FACS staining buffer and incubated with APC-conjugated streptavidin (1:600) (Thermo Fisher Scientific) in FACS staining buffer for 30 min. BD FACSCantoII flow cytometer was used for fluorescence signal acquisition. Irrelevant antibodies were used as a control. Background fluorescence signal from ACE2-HEK293T cells stained with APC-streptavidin conjugate was used for gating.

### FACS-based autoreactivity assay

Human HEp-2 cells were detached from the plastic using Versene solution, washed three times with PBS, and fixed with 2% paraformaldehyde for 10 min on ice. Saponin (0.05%) was then added to permeabilize the cells. Primary antibodies specific for Actin (ab3280, Abcam) and BIP (ab21685, Abcam) were used as positive controls. All subsequent steps were done in FACS staining buffer supplemented with saponin. Specifically, cell suspensions were incubated with antibodies (5 μg/mL) for 30 min, washed extensively, which was followed by the addition of APC-conjugated donkey-anti-human IgG (1:600) (Jackson ImmunoResearch), goat-anti-rabbit-Alexa488 (1:600, Thermo Fisher Scientific), or goat-anti-mouse-Alexa488 (1:600, Thermo Fisher Scientific). Cells to which no primary antibody was added were used to determine the level of background fluorescence. Cell suspensions were then analyzed on BD FACS Canto II cytometer. Fold increase in autoreactivity was calculated using the following formula: MFI(antibody)–MFI(background control)/MFI(background control).

### Production of SARS-CoV-2 S-pseudotyped lentiviral particles

HEK293T cells were transfected as above with a 4:6:3 molar mixture of plasmids psPAX2, pLV-EGFP, and a pCAGGS-SpikeΔ19 plasmid encoding either a WT or mutated variants of truncated SARS-CoV-2 S protein (SΔ19). Eight hours after transfection, growth medium was replaced with Opti-Mem (Gibco, Thermo Fisher Scientific) supplemented with 2.5% FBS (Gibco, Thermo Fisher Scientific). Two days later, supernatants were collected and pre-cleared by low-speed centrifugation. Next, pseudotyped lentiviral particles were concentrated by centrifugation at 20,000× *g*, 4 °C for 90 min and used in downstream assays.

### SARS-CoV-2 S-pseudotyped lentivirus neutralization assay

ACE2-HEK293T cells stably expressing human ACE2 were seeded at a density of 30,000 cells/well in a 96-well plate on the day of neutralization assay. Antibodies were serially diluted in Opti-MEM + 2.5% FBS in twofold steps to concentrations ranging from 0.5 ng/mL to 1 µg/mL and co-incubated with 20,000 S-pseudotyped lentiviral particles for 30 min at 37 °C in a volume of 100 µL. Twofold serum dilutions were prepared (range 1:80–1:2560) and processed similarly. Antibody (serum)/S-pseudotyped lentivirus mixture was added to ACE2-HEK293T cells and the plate was returned to CO_2_ incubator. 72 h following transduction, the percentage of transduced (GFP^+^) cells was measured in the cultures using flow cytometry. The half-maximal inhibitory concentration (IC_50_) or inhibitory dilution (ID_50_) was determined by non-linear regression as the concentration of antibody/serum dilution that neutralized 50% of the pseudotyped lentivirus. Data from two independent experiments were used.

### SARS-CoV-2 isolates

Two distinct clinical isolates of SARS-CoV-2 were used in our work. hCoV-19/Russia/Moscow-PMVL-12/2020 (EPI_ISL_572398), which belongs to the B.1.1.4 lineage and carries three S mutations (D614G, S686del, and V687I) neither of which map to the RBD, as well as a single amino acid deletion in the S1/S2 cleavage loop, was used for in vitro neutralization assays. A second isolate, SARS-CoV-2/human/RUS/Nsk-FRCFTM-1/2020 (EPI_ISL_481284) characterized by a single D614G substitution in the S protein (lineage B.1) was used for in vivo studies in hamsters.

### SARS-CoV-2 neutralization assay

Microculture virus neutralization assay was performed as described by Nurtop and colleagues^70^ in 96-well plates (Costar) using VeroE6 cells grown in DMEM + 5% FCS as the targets. FCS supplementation was reduced to 1% in the maintenance medium. Equal volume of serially diluted monoclonal antibodies were mixed with the viral supernatant (100 TCID_50_ (50% Tissue Culture Infectious Dose)/100 μL) and left at 37 °C for 1 h. Virus/antibody mixes (100 μL) were then transferred into wells with a monolayer of VeroE6 cells. On days 5–7 post infection, wells were visually screened under an inverted microscope. IC_100_ was defined as the lowest concentration of antibody (or the highest dilution of the serum) that provided complete protection against 100 TCID_50_ in 100 μL. Viral titers were verified for each experiment.

### Assessment of antibody concentration via BLI

Concentration of purified antibodies or antibodies present in crude supernatants of transfected cells was measured on an OctetK2 instrument (ForteBio) using Protein A Calibrator Set (cat #18-1031) and ProA biosensors (cat #18-5010). Each antibody dilution was run in duplicate. Calibration curve was plotted based on 8 standard IgG concentrations (1, 3, 10, 30, 100, 300, 500, 700 μg/mL) in at least two replicates.

### BLI measurements of *K*D, *K*on, and *K*off for purified antibodies

Measurement of binding kinetics for antibody/ RBD-His6 was performed on Octet K2 instrument (ForteBio) using NTA biosensors (cat #18-5101). RBD-His6 (30 μg/mL) was immobilized on the sensor chip. Irrelevant 6× His-tagged Fn3 protein (30 μg/mL) of similar molecular weight was immobilized on the control sensor. Next, the proteins were irreversibly immobilized on the sensors via EDC/NHS coupling, according to the manufacturer’s instructions. To characterize the *K*D values for the selected monoclonal antibodies and RBD-His6, single-point (26.7 nM concentration of RBD-His6 was chosen) measurements were done. Full Local Fitting, Bivalent model, R2 > 0.9, Χ2 < 3 was used for data processing. Nine leading antibody candidates (iB3, iB6, iB9, iB12, iB13, iB14, iB15, iB19, and iB20) were subjected to a more detailed kinetic characterization using several concentration datapoints. For this purpose, we used AHC biosensors (cat #18-5060) and immobilized the antibodies of interest or the control irrelevant HIV-specific antibody gl-VRC01 (75 μg/mL). Loaded sensors were run against a panel of six RBD concentrations (6.25 nM, 12.5 nM, 25 nM и 50 nM, 100 nM, 200 nM). Data processing was based on the Full Global Fitting, Bivalent model, R2 > 0.9, Χ2 < 3 as well as following steps: Baseline 30 s, Loading 300 s, Baseline2 60 s, Association 600 s, Dissociation 1500 s, Regeneration 3 × 5 s.

### Epitope binning (competition assay) via BLI

NTA biosensors (cat #18-5101) were used for epitope binning. 14 μg/mL RBD-His6 was loaded on the sensor. Ab1 and Ab2 were added at 75 μg/mL (500 nM) concentration. The following 6-step scheme was used: Baseline 60 s, Loading 200 s, Baseline2 30 s, Saturation Ab1 600 s, Competing Ab2 600 s, Regeneration in Glycine-HCl, pH 1.7, 5 × 3 s.

### In vivo protection assay in hamster model

Female Syrian hamsters housed in the SPF animal facility of the Institute of Cytology and Genetics SB RAS (Novosibirsk) were purchased for animal studies. Animals were weighted and the values ranged from 75.5 to 108.5 g. In the prophylactic scheme, seven groups of animals (*n* = 6 each) were intraperitoneally (i/p) administered with 10 mg/kg iB12 (group 1), 1 mg/kg iB12 (group 2), 10 mg/kg 1:1 mixture of iB6 and iВ20 (group 3), 1 mg/kg mix of iB6 and iВ20 (group 4), 10 mg/kg total human IgG (group 5, negative control) 24 h before infection (–1 dpi). Untreated animals (group 6) were used as an additional baseline control. Next day, (0 dpi) SARS-CoV-2 was given intranasally (50 μL/nostril) at a total dose of 1.3 × 10^3^ plaque-forming units. In the therapeutic regimen, six hamsters were infected as above and administered iB12 (10 mg/kg, i/p) 6 h post-infection (group 7). Animals were monitored for any signs of distress and weighed daily. In each group, three animals were euthanized on 5 dpi. Lungs were extracted for quantification of viral load and pathology analysis; blood samples were collected to measure the levels of human IgGs in the serum. Experiment to address the protective activity of iB14 nAb was similar by design, but included extended groups (6–7 animals) and an additional therapeutic timepoint at 24 h.

### ELISA of human IgG levels in hamster sera

Levels of injected human IgG antibodies in the sera of infected hamsters were assessed by ELISA. 96-well microtiter plates (Greiner Bio-One) were pre-coated with 2 μg/mL of mouse anti-human IgG monoclonal antibodies overnight at 4 °C. After blocking with 2% milk in PBS, hamster sera diluted as 1:200 (animal groups 1, 3, 5, 7) or 1:20 (groups 2, 4, 6) were added to the wells and incubated for 1 h at room temperature. The plates were then washed five times with PBST and mouse monoclonal anti-human IgG-HRP (1:12,000) was added. After incubation for 1 h at room temperature, the plates were washed and developed with TMB substrate. The reaction was stopped by the addition of 0.5 M H_2_SO_4_ and OD_450_ was measured on Multiskan FC (Thermo Scientific) plate reader.

For the iB14 efficacy in vivo experiment, hamster sera were processed as above. Dilutions of 1:200 and 1:20 were used for animals injected with 10 mg/kg and 1 mg/kg iB14 (or control total human IgG), respectively.

### Assessment of viral load by RT-qPCR

Half of the left lobe of the hamster lung was weighted, placed into LIRA solution (Biolabmix) and frozen. The samples were then thoroughly homogenized and RNA was isolated according to instructions of the manufacturer. RevertAid cDNA synthesis kit (Thermo Scientific) was used to convert 1 mg total RNA into cDNA (random hexamers were used). RT-qPCR using a 1:10 dilution of cDNA as a template was set up. Two sets of primers specific for SARS-CoV-2 *RdRp* and *E* genes have been described^71^, and are known to quantify the levels of genomic or genomic+subgenomic RNAs in the sample. Primers detecting hamster *Rpl18* transcripts^72^ were used for inter-sample normalization. Standard curve obtained by spiking total hamster lung cDNA with the known copy number of plasmid encoding E, was used to establish the minimum detection threshold of the assay as well as to convert the ΔCt values into copies of the virus. Each assay was performed in triplicate with two technical replicates, and each assay included no-template negative controls.

### Hamster lung histopathology grading

Paraffin-embedded lung sections were stained with haematoxylin and eosin (H&E) and analyzed by a trained histologist (MAG) who was masked as to the sample origin. Lung injury severity scores were assigned as follows. Normal: lung structure is nearly identical to that of the control, non-infected animals. Mild: features of mild interstitial pneumonia, lumena of alveoli are narrowed, singular perivascular swellings, alveolar septae are visually thickened. Moderate: pronounced desquamation of the epithelial lining and perivascular swellings of large vessels. Severe: advanced interstitial pneumonia, multiple interstitial swellings, lung structure damaged, large areas of atelectasis and emphysema, necrotic foci, leukocytic infiltration, multiple haemorrhages.

### Statistical analyses

Statistical analysis of the significance of differences between groups was performed in GraphPad Prism 6: ordinary one-way ANOVA (multiple comparisons) for the viral load analysis (Fig. 6b), and two-way ANOVA for the weight change analysis (Fig. 6a).

## Supplementary information

Supplementary Information
