# Supplementary material for: Isolation of a panel of ultra-potent human antibodies neutralizing SARS-CoV-2 and viral variants of concern
Source: Cell Discov. 2021 Oct 19;7:96. doi: 10.1038/s41421-021-00340-8 (PMC8526700; doi:10.1038/s41421-021-00340-8)
Supplement: Supplementary file 1 — Supplementary Information [file 41421_2021_340_MOESM1_ESM.pdf]

Supplementary Information

This PDF file includes:

Figs. S1-S9
Tables S1-S3

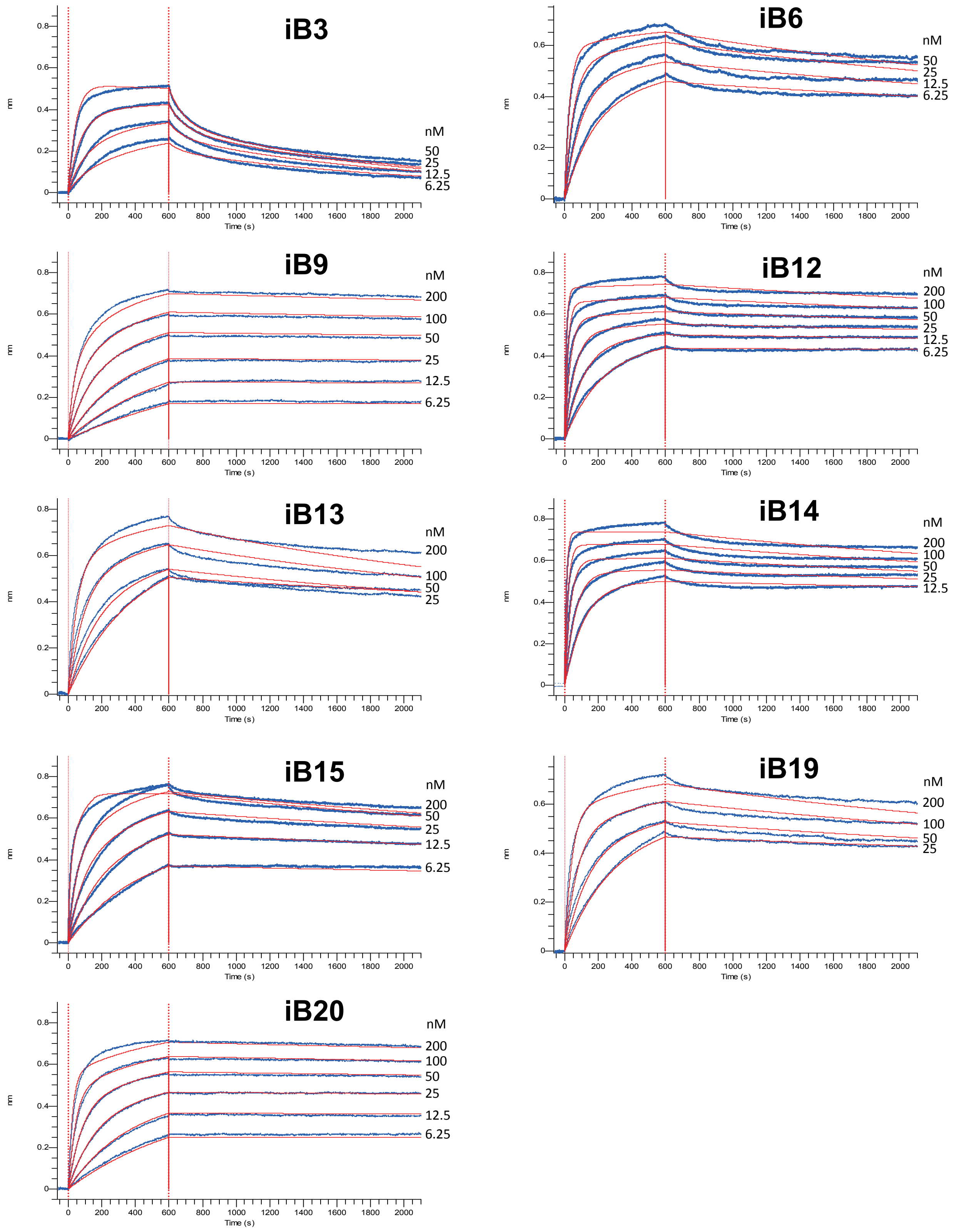

Supplementary Fig. S1. Biolayer interferometry analysis of interactions between iB antibodies (6.25-200 nM) and SARS-CoV-2 RBD.

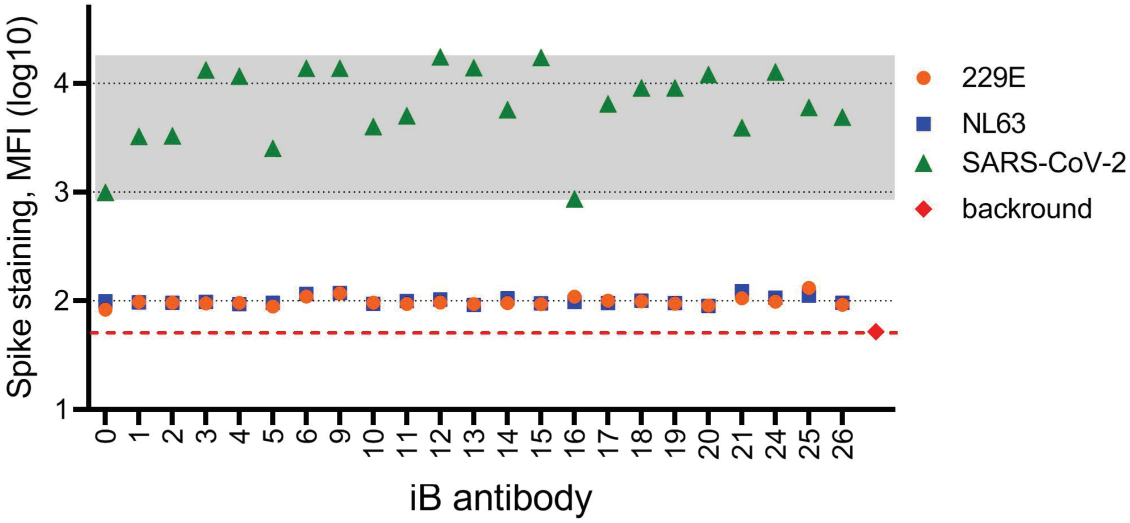

Supplementary Fig. S2. SARS-CoV2 RBD-specific antibodies (iB) selectively recognize surface-expressed Spike from SARS-CoV-2, but not that of the distantly related alphacoronaviruses HCoV-229E or HCoV-NL63.

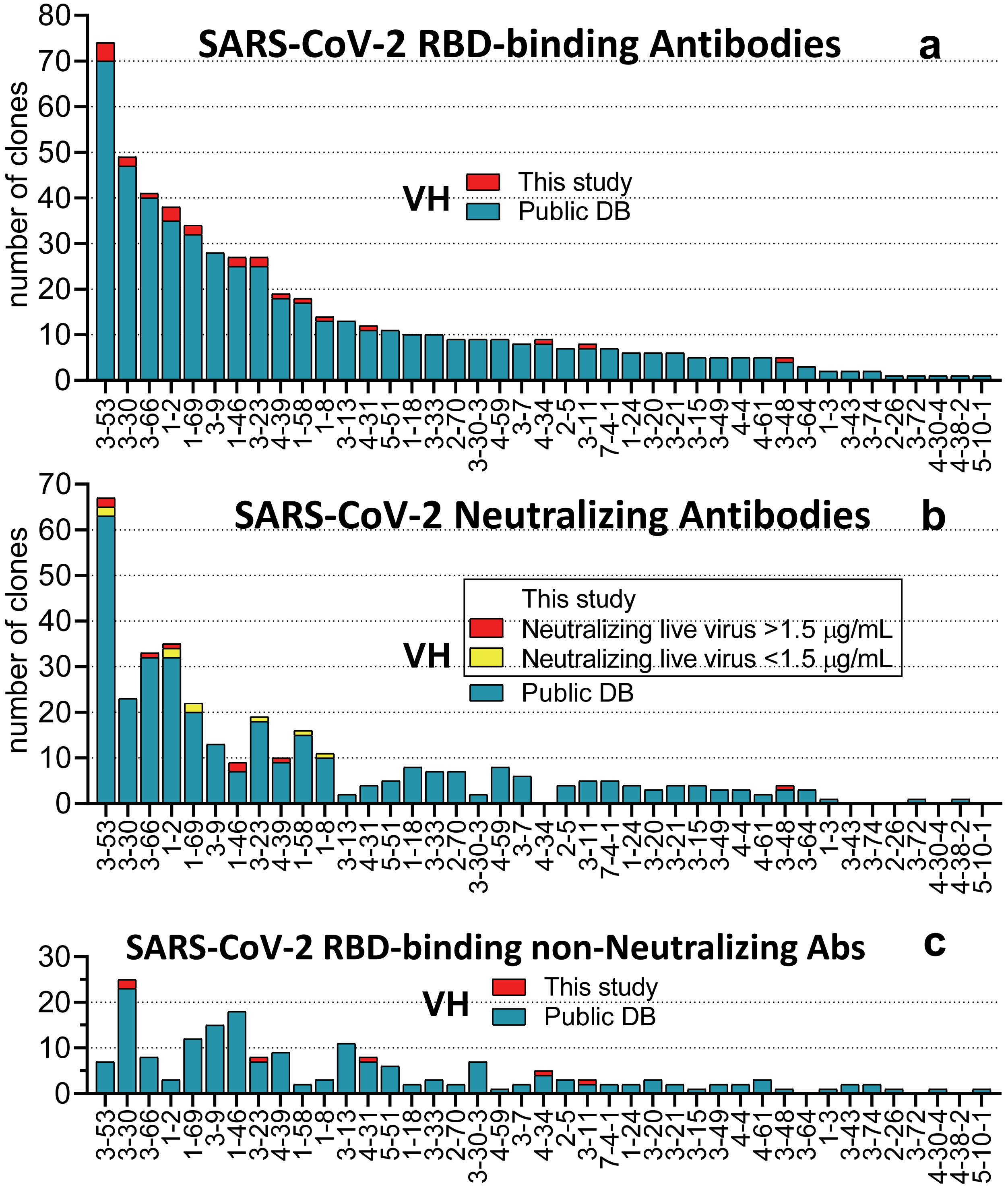

Supplementary Fig. S3. V usage in heavy chains (VH) of anti-RBD antibodies obtained in this study (red and yellow) and from Cov_AbDab database (cyan, http://opig.stats.ox.ac.uk/webapps/covabdab/). Either all RBD-binding (a), RBD-binding neutralizing SARS-CoV-2 (b) or non-neutralizing (c) VHs are shown.

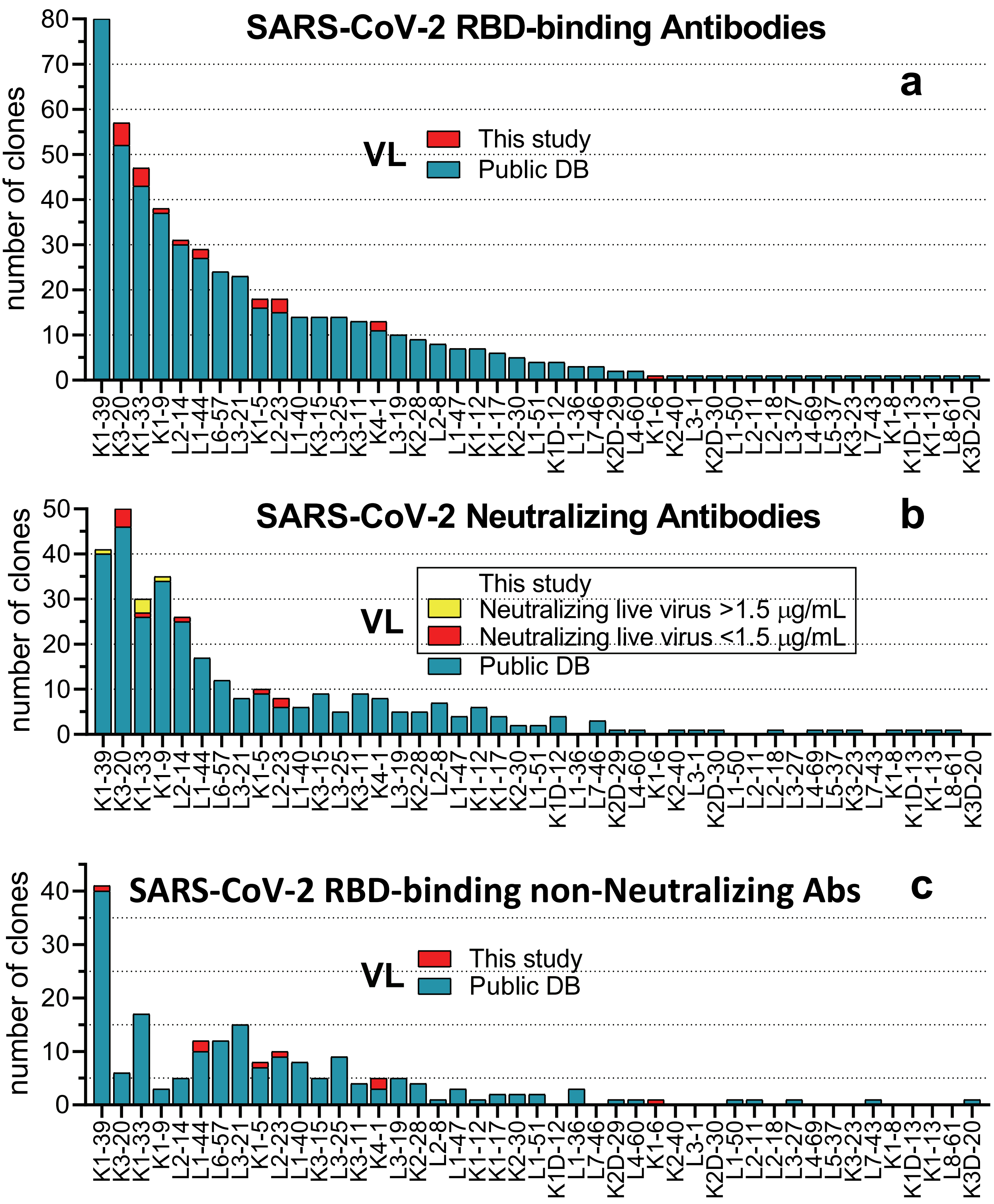

Supplementary Fig. S4. V usage in light chains (VL) of anti-RBD antibodies obtained in this study (red and yellow) and from Cov_AbDab database (cyan http://opig.stats.ox.ac.uk/webapps/covabdab/). Either all RBD-binding (a), RBD-binding neutralizing SARS-CoV-2 (b) or non-neutralizing (c) VLs are shown.

	ACE2-RBD block.	S-prot. stain.	Auto-react.	Neutr.	*K*D	SHM	CRD3 H	CDR3 L
ACE2-RBD blocking	1	0,157	0,176	**0,003**	0,061	0,496	0,905	0,742
S-protein staining	-0,37	1	0,06	**0,006**	0,771	0,233	0,232	**0,017**
Autoreactivity	0,36	-0,48	1	0,339	0,819	0,903	0,880	0,195
Pseudovirus neutralization	**0,7**	**-0,65**	0,26	1	0,803	0,135	0,388	0,689
*K*D	0,48	0,08	-0,06	0,07	1	0,362	**0,025**	0,692
SHM (aa)	-0,18	0,32	-0,03	-0,39	0,24	1	**0,023**	0,104
CRD3H length	-0,03	0,32	0,04	-0,23	**0,56**	**0,56**	1	0,182
CDR3L length	-0,09	**0,58**	-0,34	-0,11	0,11	0,42	0,35	1

Supplementary Fig. S5. Correlation heatmap matrix for the parameters of interest. Correlation coefficients (below the diagonal) are shown, and significant values (above the diagonal) are highlighted boldface. The matrix is based on values at Supplementary Tables S2 and S3.

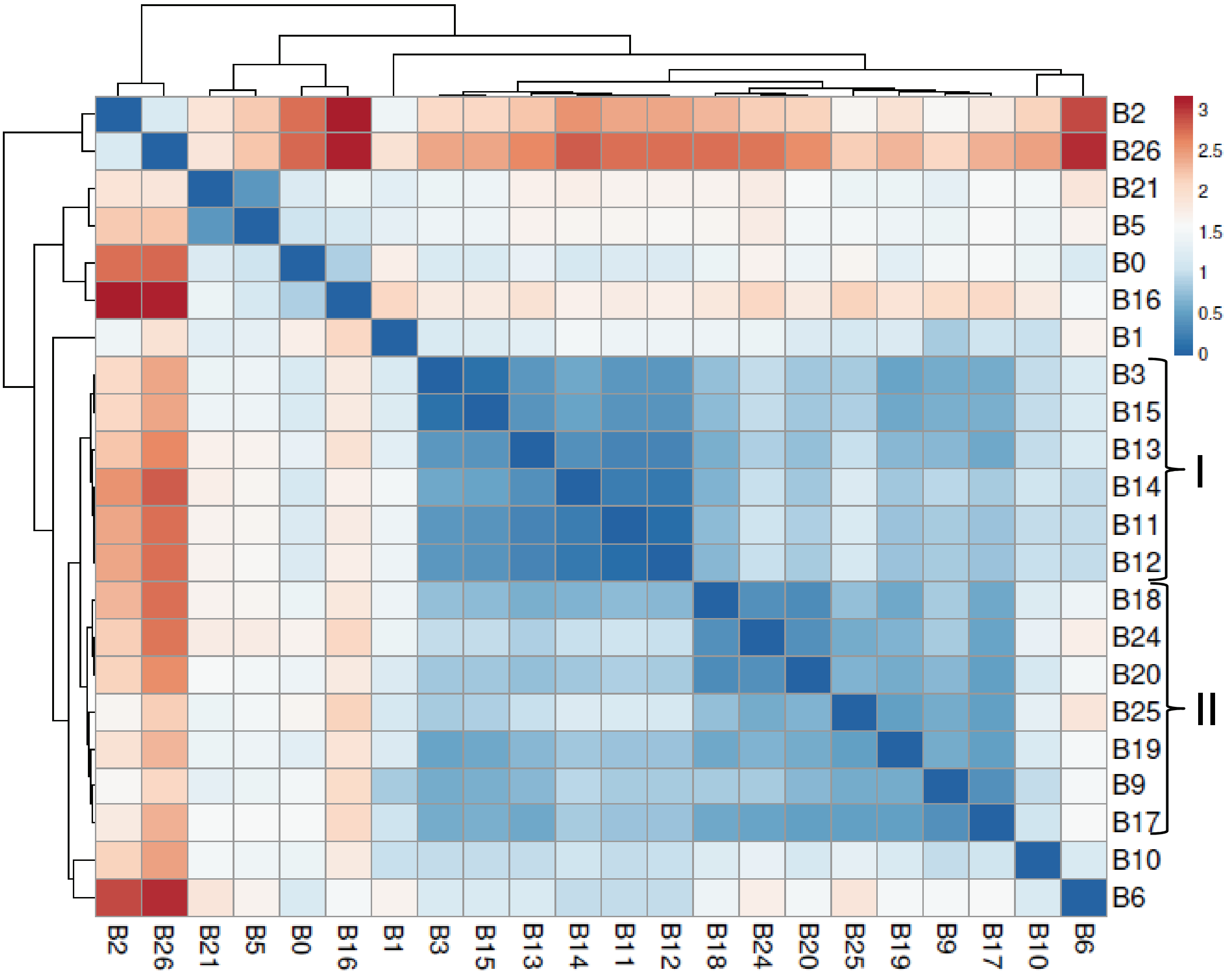

Supplementary Fig. S6. Heatmap displaying pairwise competition between the iB-series of antibodies in the BLI assay. Rows and columns are hierarchically clustered. The heatmap was made with Clustvis tool (https://biit.cs.ut.ee/clustvis/), (Metsalu and Vilo. Nucleic Acids Research, 43(W1):W566–W570, 2015. doi: 10.1093/nar/gkv468).

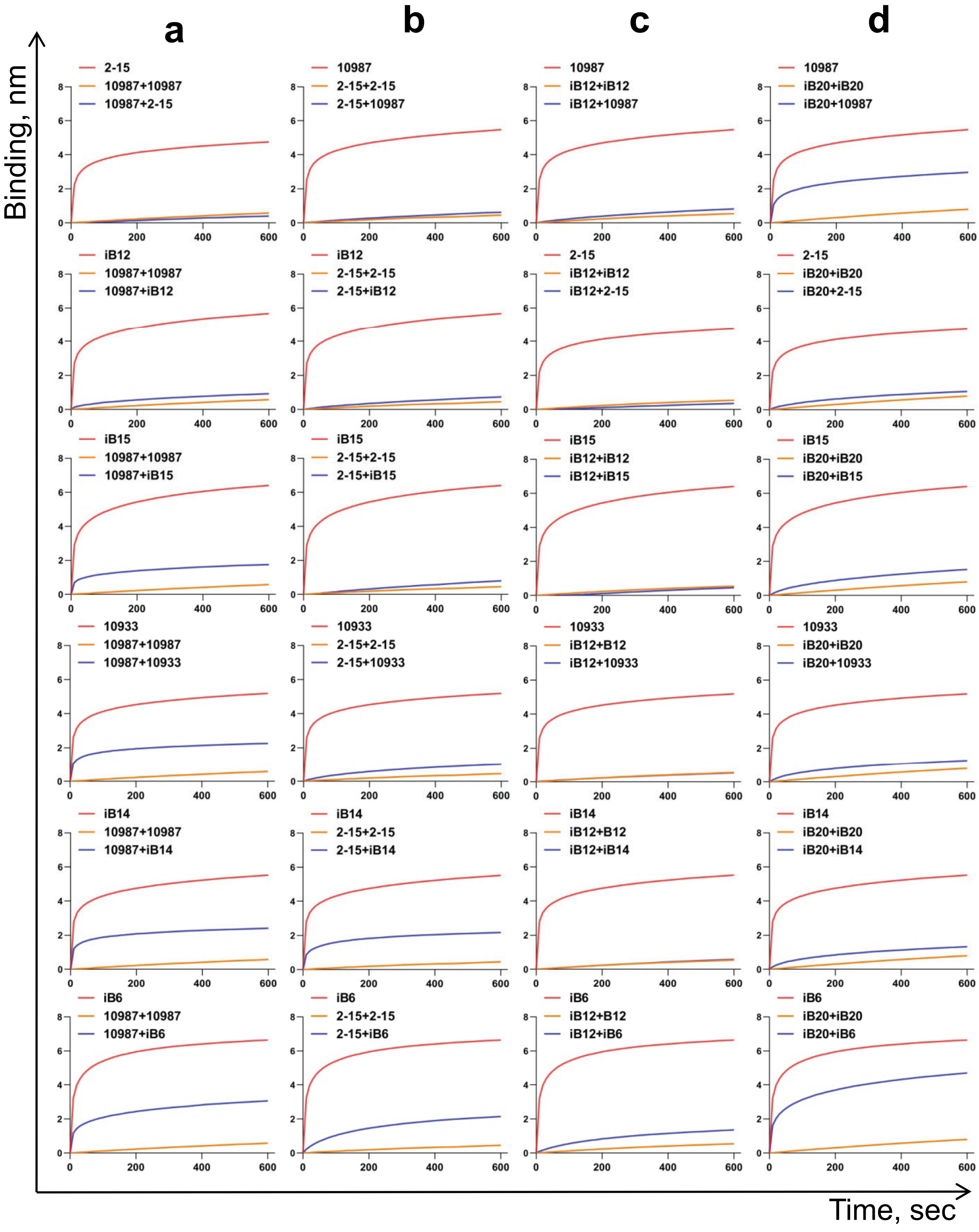

Supplementary Fig. S7. Competition of nAb pairs for binding to the RBD domain of SARS-CoV-2 as determined by BLI. Different combinations with REGN100987 (**a**), COVA2-15 (**b**), iB12 (**c**), and iB20 (**d**) are shown.

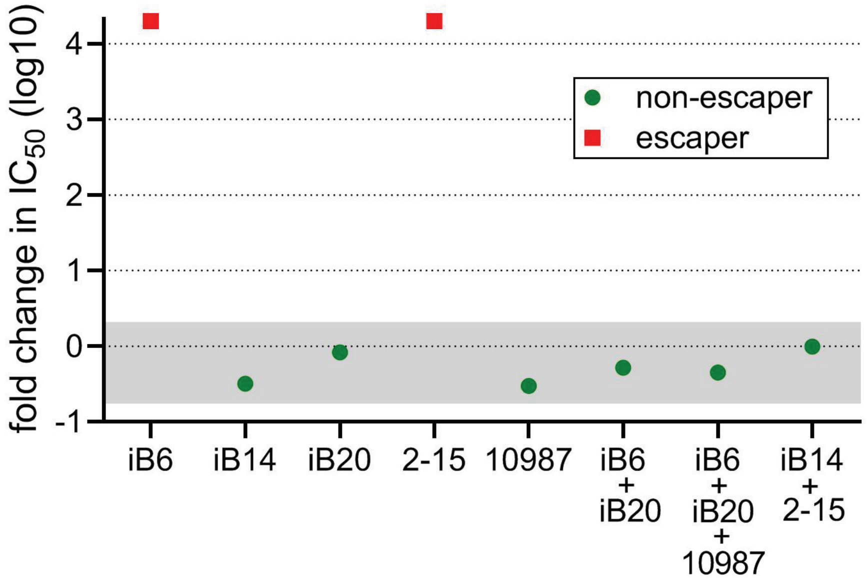

Supplementary Fig. S8. Neutralization activity of select neutralizing antibodies (nAbs) against S^{KEN}–pseudotyped lentiviruses. Fold change in IC_{50} compared to the neutralization activity against the ancestral, wild-type Spike variant is shown on the y axis. Grey-shaded area indicates the range of 5-fold increase or decrease in IC_{50} that we arbitrarily consider inconsequential for the nAb potency.

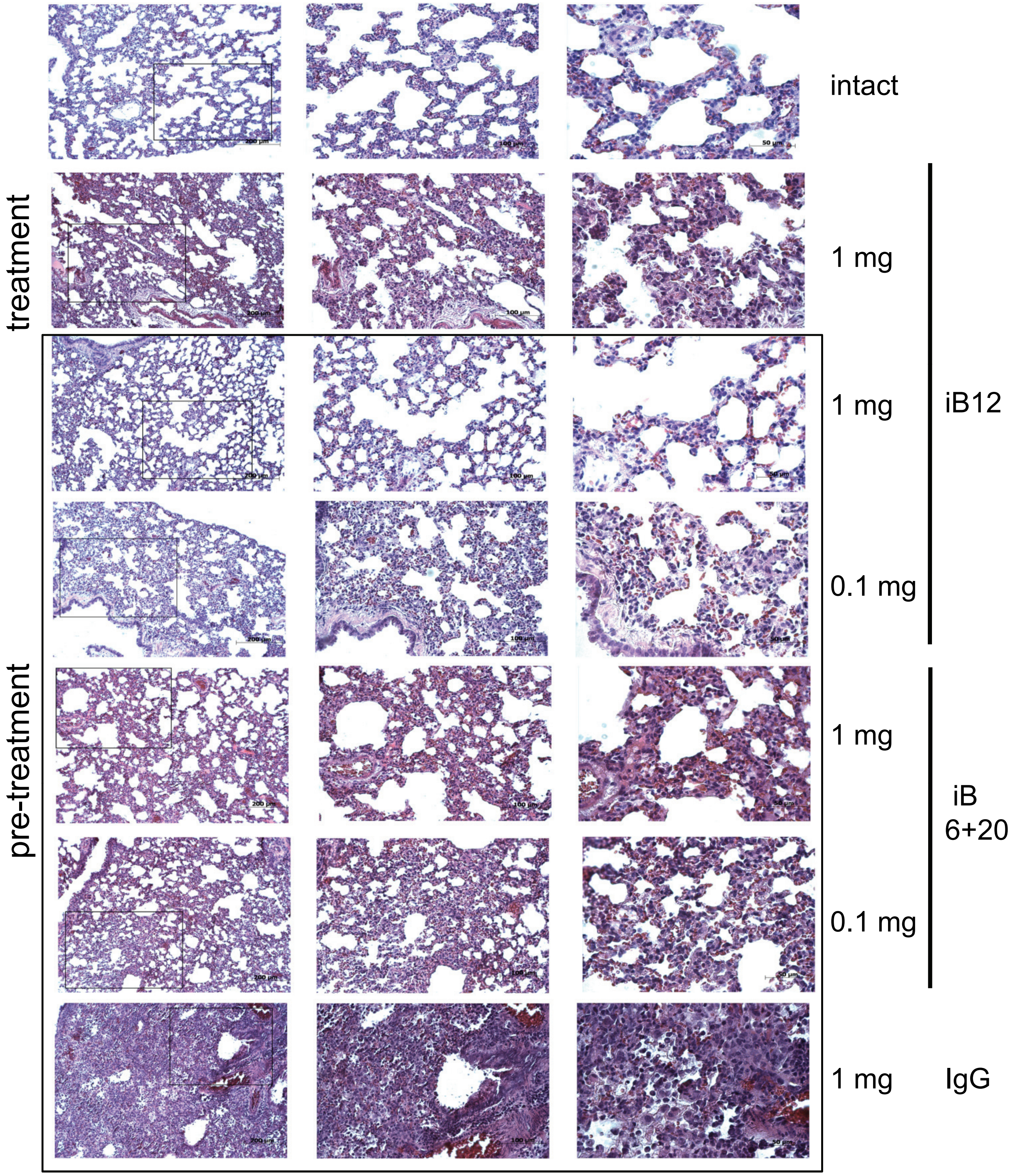

Supplementary Fig. S9. Histopathological analysis of SARS-CoV-2 infection in hamster lungs. Representative images (haematoxylin and eosin staining of lung sections from each animal group at day 5 post infection) are shown. Zoom-in images are shown in the central and right columns.

Sample ID	$CD19^+IgG^-RBD^{hi}$		$CD19^+IgG^-RBD^{med}$		$CD19^+IgG^+RBD^{hi}$		$CD19^+IgG^+RBD^{med}$		$CD19^+IgG^+$		B cells
	count	%	count	%	count	%	count	%	count	%	count
C33	4	0.069	5	0.087	4	0.069	36	0.624	2979	51.65	5767
C34	178	0.169	292	0.277	95	0.090	175	0.166	9954	9.45	105384
C35	32	0.027	387	0.327	38	0.032	349	0.295	19697	16.66	118259
C36	3	0.030	23	0.233	9	0.091	37	0.375	1714	17.38	9861

Supplementary Table S1. Distribution of $CD19^+$ B cell subpopulations as assayed by FACS (Fluorescence-Activated Cell Sorting) staining of donor B lymphocytes.

Ab	ACE2-RBD blocking[1]		Spike staining[2]		Autoreactivity[3]		Virus Neutralization		Affinity[6]		
	Median MFI	Protection effect in %	Mean MFI	In % from best Ab	Mean MFI	OBF, fold	Pseudo[4] IC_{50}, ng/mL	Live[5] IC_{100}, ng/mL	*K*D, nM	*K*on, $M^{-1}sec^{-1}$	*K*off, sec^{-1}
iB0	6674	0.44	996	5.36	1110	5.53	ND	ND			
iB1	6502	3.05	3260	18.22	273	0.61	ND	50000			
iB2	5029	25.43	3299	18.44	401	1.36	ND	ND			
iB3	4528	33.04	13270	75.08	369	1.17	13±2	16	77.80±1.32	$1.19±0.01 \times 10^5$	$9.28±0.15 \times 10^{-3}$
iB4	5224	22.47	11619	65.70	313	0.84	>500	25000			
iB5	7317	-9.33	2533	14.09	411	1.42	ND	ND			
iB6	5103	24.31	13812	78.16	608	2.58	30±11	125	0.76±0.01	$2.42±0.01 \times 10^5$	$1.85±0.02 \times 10^{-4}$
iB9	351	96.49	13741	77.76	343	1.02	13±4	500	0.99±0.02	$3.67±0.03 \times 10^4$	$3.65±0.09 \times 10^{-5}$
iB10	4133	39.04	4027	22.58	244	0.44	ND	ND			
iB11	1774	74.87	5057	28.43	927	4.45	>200	6000			
iB12	1195	83.67	17493	99.07	306	0.80	8±3	4	0.47±0.01	$1.77±0.01 \times 10^5$	$8.33±0.09 \times 10^{-5}$
iB13	1004	86.57	13959	78.99	335	0.97	>200	500	8.62±0.21	$3.02±0.03 \times 10^4$	$2.60±0.06 \times 10^{-4}$
iB14	374	96.14	5744	32.33	311	0.83	5±1	8	0.73±0.01	$1.83±0.01 \times 10^5$	$1.34±0.01 \times 10^{-4}$
iB15	NA	88.00	17337	98.18	329	0.94	11±2	2	1.09±0.01	$1.25±0.01 \times 10^5$	$1.36±0.01 \times 10^{-4}$
iB16	6616	1.32	869	4.641	668	2.93	ND	50000			
iB17	3070	55.19	6503	36.64	319	0.88	>1000	25000			
iB18	1953	72.16	9101	51.40	326	0.92	80±30	1500			
iB19	NA	52.00	9123	51.53	394	1.32	>400	250**	4.73±0.01	$3.76±0.03 \times 10^4$	$1.78±0.04 \times 10^{-4}$
iB20	378	96.08	12064	68.23	296	0.74	19±4	500	0.57±0.01	$5.90±0.03 \times 10^4$	$3.35±0.08 \times 10^{-5}$
iB21	5191	22.97	3917	21.95	1781	9.48	ND	ND			
iB24	2080	70.23	12774	72.26	365	1.15	>150	12500			
iB25	3777	44.45	6006	33.82	397	1.34	>1500	25000			
iB26	5114	24.14	4885	27.45	480	1.82	ND	ND			
REGN10933	NT	NT	5384	30.29	NT	NT	6.9±0.8	4	3.37*	3.00×10^6*	1.01×10^{-2}*
REGN10987	NT	NT	9917	56.04	314	0.85	9.3±2.4	16	45.23*	8.07×10^5*	3.65×10^{-2}*
COV2-2504	NT	NT	16318	92.39	NT	NT	9.4±1.2	2	NA	NA	NA
COVA2-15	NT	NT	17657	**100**	NT	NT	10.5±1.0	8	NA	NA	NA
glVRC01	6028	10.25									
RBD only	6703	**0**									
background	120		52	**0**	170	**0**					

Supplementary Table S2. Characteristics of the iB-series and published SARS-CoV-2 RBD-specific Abs. ACE2-RBD blocking[1] was tested by adding the antibody/RBD-biomixture to ACE2-HEK293T cells. Failure of the antibody to prevent RBD-bio binding to ACE2 was revealed by FACS (Fluorescence-Activated Cell Sorting). Protection effect was calculated as $(1 - (MFI_{antibody} - MFI_{background})/(MFI_{RBD\ only} - MFI_{background})) \times 100\%$. Spike staining[2] was analyzed by flow cytometry analysis of Spike-expressing HEK293T cells incubated with the antibodies. Autoreactivity[3] and virus neutralization scores[4,5] were measured as described in the Materials and Methods. Affinities[6] were determined either with bio-layer interferometry or extracted from the literature data[*]. MFI – Mean Fluorescence Intensity, NT – not tested, ND – not detected, NA – not available.

iB	VH gene	VL gene	Sample ID	CDR length, aa		VH SHM				VL SHM			
				VH	VL	nt subst count	nt GL %	aa subst count	aa GL %	nt subst count	nt GL %	aa subst count	aa GL %
0	VH3-30	VK1-39	C34	20	9	1	99.7	0	100	0	100	0	100
1	VH3-48	VL1-44	C34	20	11	13	95.6	8	**91.7**	3	99	2	98.0
2	VH4-34	VK4-1	C34	17	9	23	**92.2**	10	**89.7**	9	97	6	**94.0**
3	VH1-2	VL2-23	C34	28	10	3	99	3	96.9	4	98.6	4	95.9
4	VH1-2	VK1-33	C34	14	11	1	99.7	1	99.0	0	100	0	100
5	VH4-31	VL2-23	C34	15	9	3	99.0	2	98.0	5	98.3	4	95.9
6	VH1-69	VK1-5	C34	14	9	4	98.3	4	95.9	1	99.7	1	98.9
9	VH1-69	VK3-20	C34	16	9	3	99.0	2	97.9	1	99.7	1	99.0
10	VH3-11	VL1-44	C34	13	11	1	99.7	1	99.0	2	99.3	1	99.0
11	VH1-46	VK1-39	C34	18	9	3	99.0	2	98.0	4	98.6	2	97.9
12	VH1-2	VK1-33	C34	14	11	5	98.3	3	96.9	1	99.7	0	100
13	VH3-23	VL2-14	C34	25	12	3	99.0	2	98.0	5	98.3	4	95.9
14	VH1-58	VK3-20	C34	16	7	1	99.7	1	99.0	3	99.0	2	97.9
15	VH1-8	VL2-23	C34	24	12	4	98.6	4	95.6	3	99.0	2	97.9
16	VH4-39	VK1-5	C34	16	8	1	99.7	1	99.0	2	99.3	1	98.9
17	VH1-46	VK1-33	C34	14	10	0	100	0	100	4	98.6	3	96.8
18	VH3-53	VK3-20	C34	11	9	2	99.3	1	99.0	3	99.0	2	97.9
19	VH3-66	VK3-20	C34	11	9	1	99.7	1	99.0	0	100	0	100
20	VH3-53	VK3-20	C35	12	10	7	97.6	4	95.9	4	98.6	3	96.9
21	VH3-23	VK4-1	C35	21	10	0	100	0	100	0	100	0	100
24	VH3-53	VK1-33	C36	13	9	1	99.7	1	99.0	0	100	0	100
25	VH3-53	VK1-9	C33	11	11	2	99.3	2	97.9	4	98.6	3	96.8
26	VH3-30	VK1-6	C33	17	9	1	99.7	0	100	0	100	0	100

Supplementary Table S3. Sequence characteristics of SARS-CoV-2 RBD-specific iB antibodies. SHM – somatic hypermutation, nt/aa GL % – percent of homology to germline sequence excluding CDR3 and FR4 regions according to IMGT (www.imgt.org). SHM values above 5% and 2.5% are highlighted red and orange, respectively.
